# Radiation-induced morphea of the breast – characterization and treatment of fibroblast dysfunction with repurposed mesalazine

**DOI:** 10.1038/s41598-024-74206-w

**Published:** 2024-10-30

**Authors:** Stephan R. Künzel, Erik Klapproth, Nick Zimmermann, Susanne Kämmerer, Mario Schubert, Karolina Künzel, Maximilian Hoffmann, Stephan Drukewitz, Anne Vehlow, Jiri Eitler, Marieke Arriens, Jessica Thiel, Romy Kronstein-Wiedemann, Maximiliane Tietze, Stefan Beissert, Bertold Renner, Ali El-Armouche, Claudia Günther

**Affiliations:** 1 Institute for Pharmacology and Toxicology, Faculty of Medicine Carl Gustav Carus, Technische Universität Dresden, Dresden, Germany; 2 Institute for Clinical Pharmacology, Faculty of Medicine Carl Gustav Carus, Technische Universität Dresden, Dresden, Germany; 3 Department of Dermatology, Faculty of Medicine and University Hospital Carl Gustav Carus, Technische Universität Dresden, Fetscherstrasse 74, Dresden Dresden, Germany; 4 Institute for Transfusion Medicine, Faculty of Medicine Carl Gustav Carus, Technische Universität Dresden and DRK Blutspendedienst Nord-Ost gGmbH, Dresden, Germany; 5 Institute of Human Genetics, University of Leipzig Medical Center, Leipzig, Germany; 6 Core Unit for Molecular Tumor Diagnostics, NCT Dresden and DKFZ, Dresden, Germany; 7 OncoRay - National Center for Radiation Research in Oncology, Faculty of Medicine Carl Gustav Carus, Technische Universität Dresden, Dresden, Germany; 8 National Center for Tumor Diseases (NCT), Partner Site Dresden, German Cancer Research Center (DKFZ), Heidelberg, Germany

**Keywords:** Fibrosis, Morphea, Drug repurposing, Breast cancer, Mesalazine, Translational research, Musculoskeletal system, Inflammation, Diagnostic markers, Breast cancer, Radiotherapy

## Abstract

Radiation-induced morphea (RIM) is a rare complication of radiotherapy presenting as inflammatory fibrosis, most commonly reported in breast cancer patients. As underlying disease mechanisms are not well understood, targeted therapies are lacking. Since fibroblasts are the key mediators of all fibroproliferative diseases, this study aimed to characterize patient-derived fibroblasts to identify therapeutic targets. We studied primary human control and RIM-fibroblasts on a functional and molecular basis, analyzed peripheral blood and tissue samples and conducted, based on our findings, a treatment attempt in one patient. In RIM, we identified a distinct myofibroblast phenotype reflected by increased alpha-smooth-muscle-actin (αSMA) expression, reduced proliferation and migration rates, and overexpression of osteopontin (OPN). Our RNA sequencing identified aberrant Myc activation as a potential disease driver in RIM fibroblasts, similar to previous findings in systemic sclerosis. Treatment with the anti-inflammatory drug mesalazine reversed the myofibroblast phenotype by targeting Myc. Based on these findings, a patient with RIM was successfully treated with mesalazine, resulting in reduced inflammation and pain and tissue softening, while serum OPN was halved. The present study provides a comprehensive characterization of RIM fibroblasts, suggests a disease-driving role for Myc, demonstrates promising antifibrotic effects of mesalazine and proposes OPN as a biomarker for RIM.

**Supplementary Information:**

The online version contains supplementary material available at 10.1038/s41598-024-74206-w.

## Introduction

Radiation-induced Morphea (RIM) is a rare and potentially under-recognized complication most commonly reported in breast cancer patients after completion of radiotherapy^1^. RIM is frequently misdiagnosed for cellulitis, mastitis or cancer recurrence^1^. Therefore, an early biopsy is important in distinguishing RIM from phenotypically similar diseases. RIM typically displays a superficial lymphocytic infiltrate and increased collagen deposition in the reticular dermis possibly accompanied by eosinophilia^2,3^. Recent epidemiological data suggests that 1 out of 378 breast cancer patients^4^is affected. The clinical appearance comprises erythema, edema and tissue induration as well as shrinkage of the affected breast, physical and emotional pain, ulceration and resulting infections^5,6^. Neither dose nor fractionation of radiotherapy seem to correlate with the incidence or severity of RIM^4,5^. Although an association to autoimmune disease has been proposed^6^, the knowledge about the underlying cellular and molecular disease mechanisms remains elusive as of today. Steroids, methotrexate, phototherapy and surgery can lead to varying symptomatic relief^1^. However, as found in systemic sclerosis treatment approaches, fibrosis is a generally poorly responding symptom^7^. Thus, a cellular and molecular characterization of RIM pathophysiology to derive novel treatment strategies is warranted.

Fibroblasts have emerged in the spotlight of antifibrotic drug research, as they are key cellular regulators of the connective tissue. Fibroblasts maintain the balance between extracellular matrix (ECM) synthesis and degradation thereby contributing to ECM and tissue homeostasis^8–10^. Upon exposure to activating stimuli like radiation^11^, chemical mediators or mechanic tissue injury^12^, fibroblasts undergo a phenotypic transition towards myofibroblasts, characterized by the expression of organized α-smooth muscle actin (αSMA) microfilaments^8,9^. Myofibroblasts promote and maintain tissue fibrosis by secretion of vast amounts of ECM and a multitude of inflammatory mediators like transforming growth factor beta (TGFβ) and interleukins^13,14^.

Currently, there are numerous therapeutic efforts aiming at preventing or even reversing fibrosis, including antibody therapies, small molecules and cellular immunotherapies^15–18^. However, in order to offer new antifibrotic therapies in a timely manner, the use of an already existing, FDA or EMA approved drug with a favorable side effect profile would be optimal (drug repurposing). First evidence has indicated that systemically administered mesalazine (5-ASA), which is clinically used to treat chronic inflammatory bowel disease, is capable of stopping and partly reversing fibrosis progression in a model of induced liver fibrosis^19^. Furthermore, we recently demonstrated that mesalazine is sufficient to prevent cardiac and dermal fibrosis in vitro and in mouse models^20–23^.

Based on the functional and molecular characterization of RIM-derived fibroblasts, we identified a potentially disease-driving role for aberrant Myc activation in RIM fibroblasts. Moreover, we provide experimental as well as clinical evidence for repurposing of mesalazine as a novel treatment approach.

## Results

### Characterization of control and RIM-derived cultured fibroblasts

**Fig. 1:**
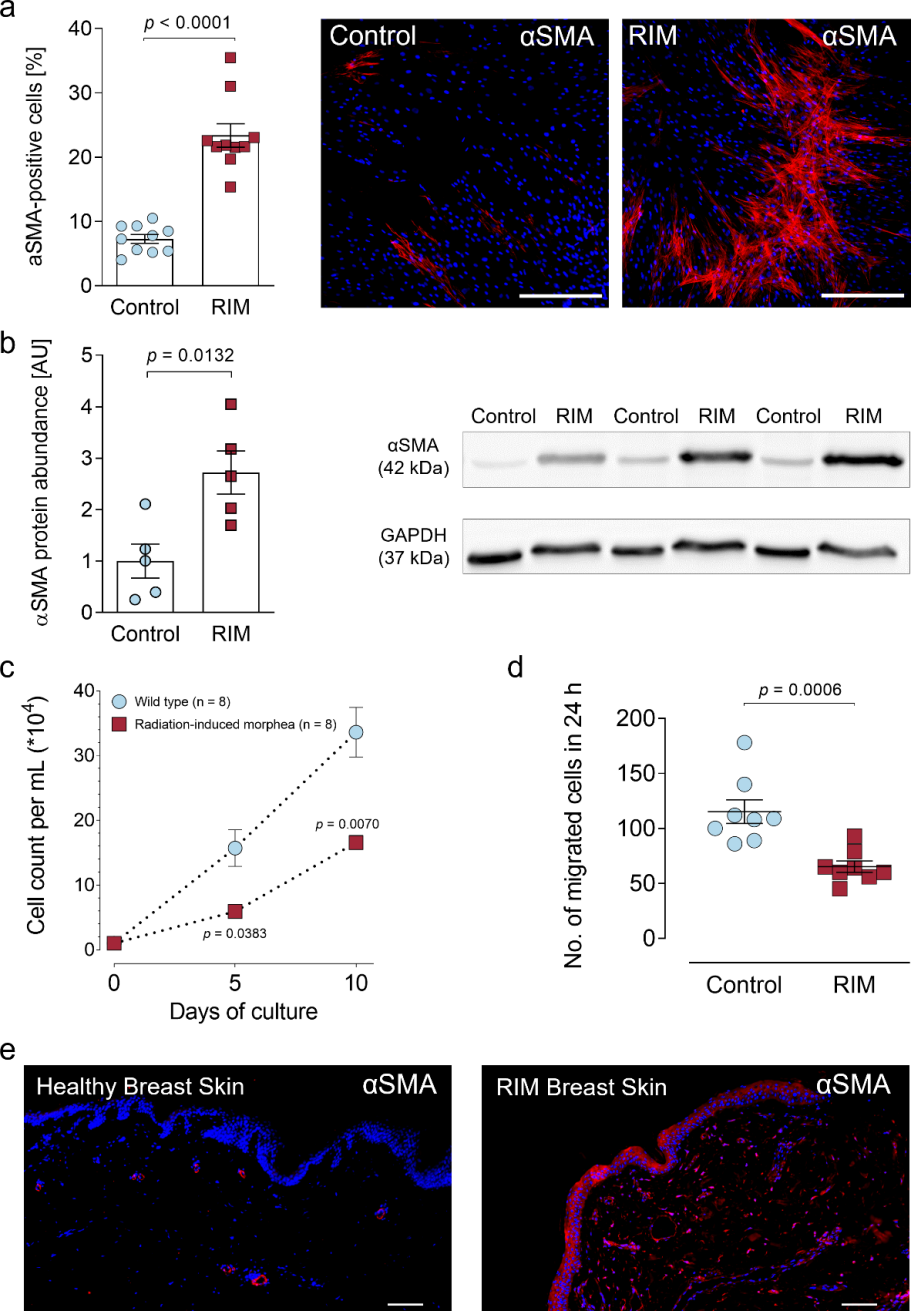
Radiation-induced morphea is accompanied by a pronounced myofibroblast phenotype.** (a)** Quantification and representative images of αSMA (alpha smooth muscle actin; red) immunofluorescence staining in primary patient-derived control and RIM (radiation induced morphea) fibroblast cultured under basal conditions (37 °C, 5% CO_2_, Dulbecco’s modified eagle medium supplemented with 10% fetal calf serum and 1% penicillin-streptomycin) for 7 d, the nuclei were stained with DAPI (blue), (*n* = 10 analyzed coverslips from *N* = 5 patients per group; results are given as mean ± SEM determined by a Mann-Whitney *U* test). The scale bars equal 200 μm. **(b)** Quantification and representative western blot of αSMA protein abundance in control and RIM (radiation induced morphea) fibroblast, normalized to GAPDH, (*n* = 5 per group; results are given as mean ± SEM determined by a Welch *t* test). **(c)** Proliferation curves of control and RIM-fibroblasts under basal conditions, (*n* = 8 from *N* = 4 patients per group, results are given as mean ± SEM determined by a Welch *t* test for each time point). **(d)** Number of migrated control and RIM-fibroblasts in a wound healing assay after 24 h, (*n* = 8 from *N* = 4 patients per group, results are given as mean ± SEM determined by a Mann-Whitney *U* test). **(e)** Exemplary immunofluorescence images of αSMA (red) in skin punch biopsies of a control and a RIM patient; the nuclei were stained with DAPI (blue). The scale bars equal 100 μm.

Primary skin fibroblasts were isolated via outgrowth^10^ from skin biopsies of 5 control and 5 RIM patients and subsequently seeded on glass coverslips for cytomorphological characterization. All cells displayed typical fibroblast morphology, reflected by a spindle shape and stellate processes (Figure S1 A). Accepted fibroblast marker proteins like vimentin, human fibroblast surface protein (hFSP), Discoidin domain-containing receptor 2 (DDR2) and collagen1 ^9,10,24^ were present in immunocytochemical analysis (Figure S1b-e). Myofibroblast differentiation is a critical step in fibrosis development^8^. Compared to control, basal expression of the myofibroblast marker αSMA as determined by immunostaining was 16.1% higher in RIM (αSMA_control_: 7.3 ± 0.68%; αSMA_RIM_: 23.4 ± 1.81%) (Fig. 1a). This finding was validated by western blot, which demonstrated significantly higher (*p* = 0.0132) αSMA protein expression in RIM (Fig. 1b). Functionally, RIM-derived fibroblasts displayed markedly reduced proliferation rates and reduced migration in wound healing assay compared to control indicating their reduced wound healing potential (Fig. 1c and d). Senescence-associated beta-galactosidase activity^25^ was not detectable in either group. Lastly, exemplary immunohistochemistry revealed diffuse tissue αSMA expression in a RIM-derived skin punch biopsy suggesting exaggerated myofibroblast presence, whereas an αSMA signal was only detectable around blood vessels in the respective control sample (Fig. 1e).

### Gene expression analysis

**Fig. 2:**
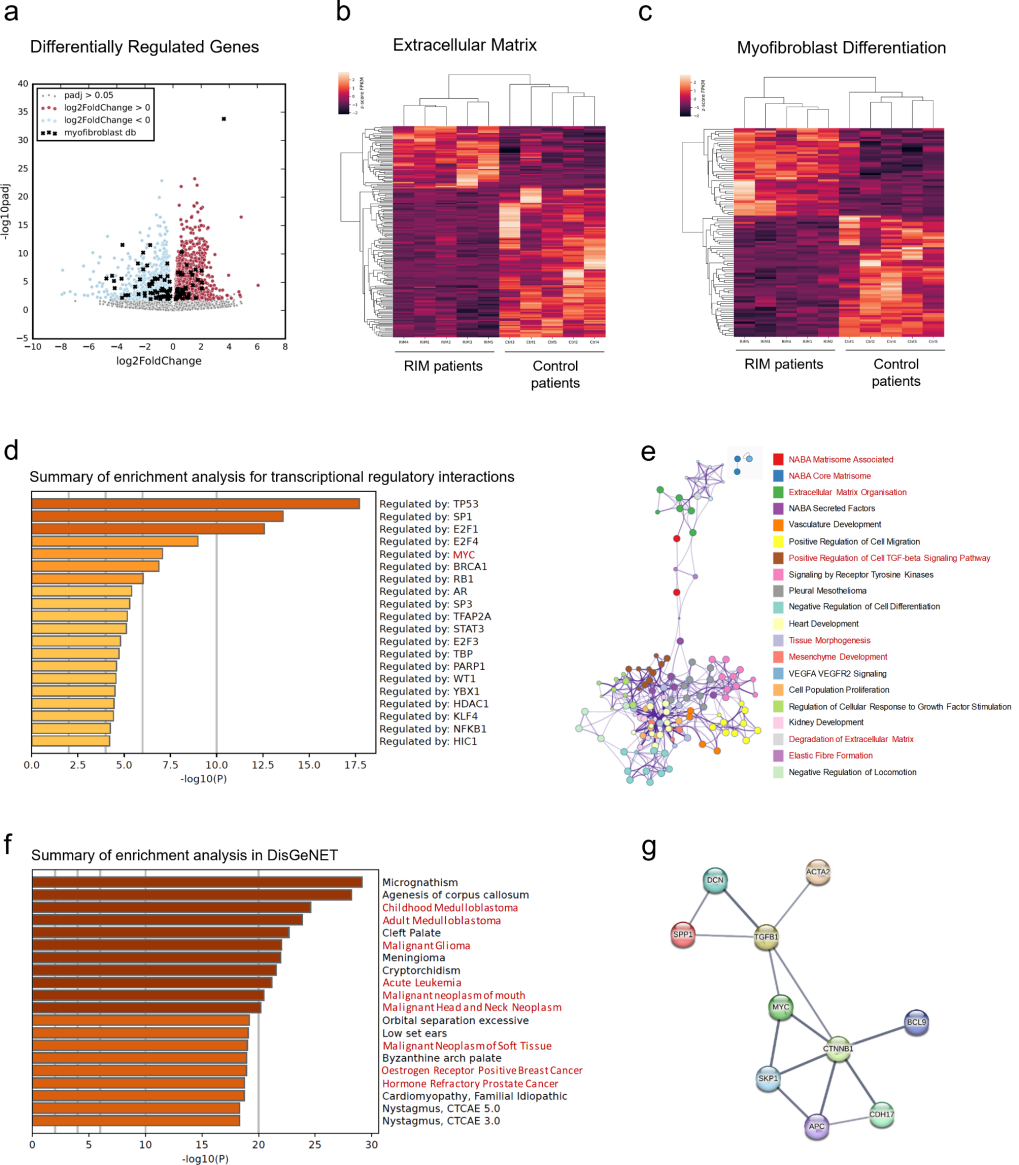
Differential gene expression analyses and FPKM value based clustered heatmaps comparing RIM samples with the control group.** a)** Deseq2 results depicted in a volcano plot. Significantly up-regulated genes are depicted in red, significantly down-regulated genes are depicted in blue. Genes which are listed in the myofibroblast differentiation (b) dataset are highlighted with a black cross. The x-axis shows the log2FoldChange and the y-axis the negative log10 value of the adjusted p-value for each gene. **b**,** c)** Expression value based clustered heatmaps of three manually selected gene sets. Clustering of RIM and control samples shows a clear separation between the two groups. Comprehensive gene lists with expression values for each gene set of interest are provided in Table S1. **d)** Metascape analysis of significantly enriched transcriptional regulatory interactions. **e)** Network of enriched terms using Cytoscape5 ^78^. Each node represents an enriched term and is colored by its cluster ID. **f)** Metascape analysis of significantly enriched disease associated pathways. **g)** Protein-protein interaction network with the fibrosis-associated genes Myc, CTNNB1, ACTA2, SPP1 based on textmining, experimental data, databases and co-expression with high confidence (0.700) using the STRING database. The line thickness indicates the strength of data support.

We performed mRNA sequencing (RNA-Seq) of primary control and RIM-derived patient skin fibroblasts to explore putative differences in gene expression with particular emphasis on myofibroblast differentiation, cytokines and extracellular matrix as well as regulatory pathways. Differential gene expression analyses revealed a total of 3641 differentially expressed genes (*p*_adjusted_ < 0.05), that split into 1792 up-regulated and 1849 down-regulated genes when comparing the RIM samples to the control group (Fig. 2a-c; Table S1). Gene sets focusing on fibrosis mechanisms for subsequent analysis were provided by GSEA^26–28^and The Harmonizome^29^. Additionally, we performed pathway analysis with Metascape to uncover the functional relevance of differentially expressed genes^30^. In line with the results of the phenotypic characterization, RIM-fibroblasts showed a significantly (*p* = 0.0095) higher expression of *ACTA2* (αSMA). We found robust expression levels of *matrix metalloproteinase* (*MMP*) *1*,* 2*,* 3* and *14* with significantly lower *MMP2* (*p* = 9.357e-05) and *MMP14* (*p* = 0.0017) expression in RIM compared to control (Fig. 2a, b; Table S1). There was no difference in the expression of *transforming growth factor beta* (*TGFβ*,* TGFB1*) (*p*= 0.1518), which is widely regarded as the “master regulator” of fibrosis^31^. However, the TGFβ pathway showed significant enrichment (Fig. 2e). In line with this finding, RIM-fibroblasts were found to have a significantly higher expression of the TGFβ downstream target *osteopontin* (*OPN*; Table S1), which is both a constituent of the extracellular matrix and a cytokine^32,33^relevant in cardiac, pulmonary, hepatic and dermal fibrosis^19,33–35^. Among known pro-fibrotic transcription factors, there was a significantly higher expression of *Myc* (*p* = 0.0028) and *β-Catenin* (*CTNNB1*, *p* = 7.4567e-06) in RIM (Table S1), consistent with previous findings in systemic sclerosis^36,37^. Enrichment Analysis of transcriptional regulatory networks confirmed significant enrichment of genes regulated by Myc (Fig. 2d). Further disease association analysis with Metascape using DisGeNET^38^ revealed enrichment of several cancer-associated pathways, in particular estrogen receptor positive breast cancer (Fig. 2f). Lastly, we used the STRING database^39^, which integrates protein-protein interactions to uncover functional and regulatory interactions, to create a concise interaction network of identified genes of interest (*Myc*,* CTNNB1*,* ACTA2*,* SPP1* (OPN)), which have been associated with fibrosis indicating indirect profibrotic roles for Myc and CTNNB1(Fig. 2g).

### OPN function in RIM

**Fig. 3:**
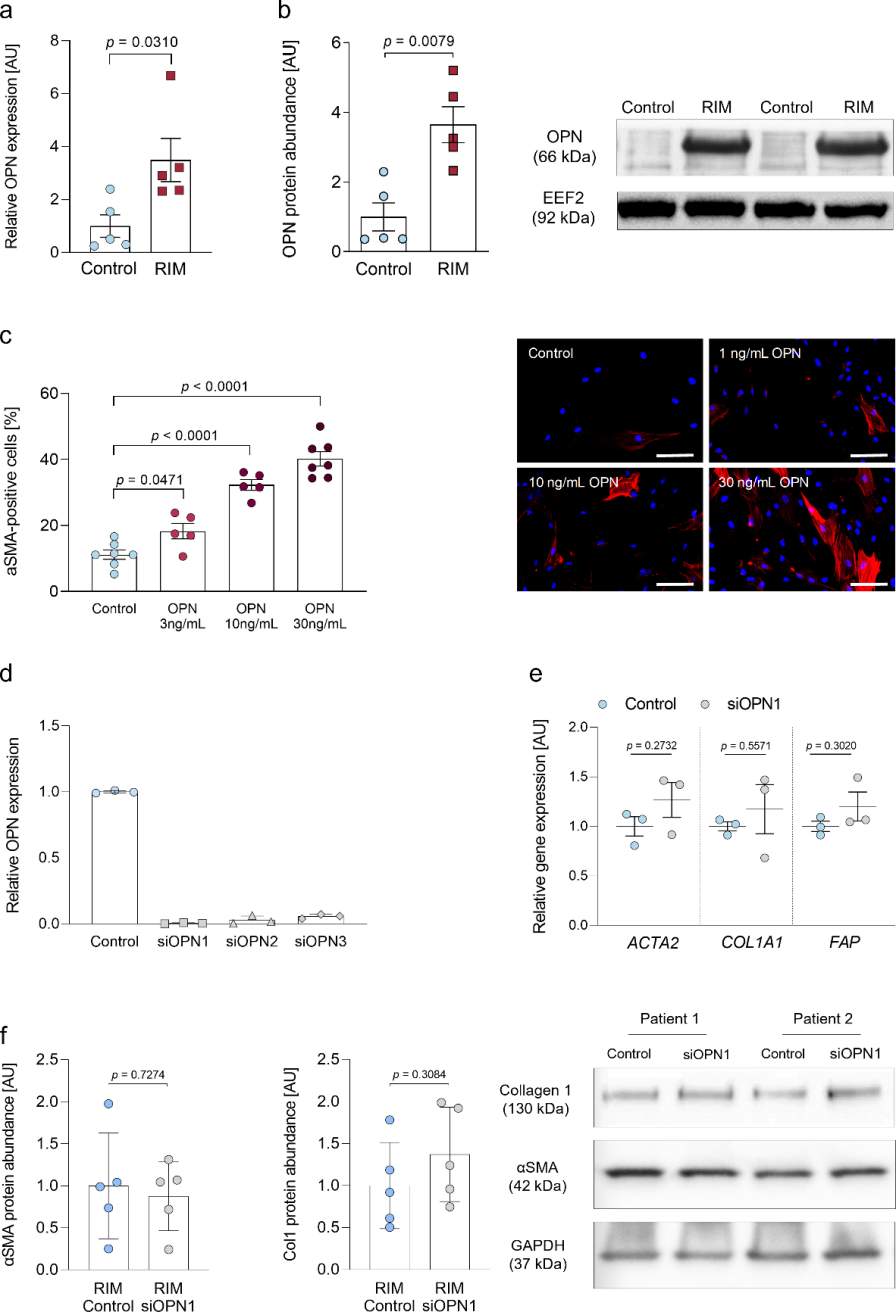
Characterization of OPN function in RIM.** (a)** Expression of *OPN* mRNA (qPCR) normalized to *EEF2* (Eukaryotic translation elongation factor 2) as housekeeping gene in control and RIM-fibroblasts, (*n* = 5 per group; results are given as mean ± SEM determined by a Mann-Whitney *U* test). **(b)** Quantification and representative western blot and of OPN protein abundance in control and RIM fibroblast, normalized to EEF2 as housekeeping protein, (*n* = 5 per group; results are given as mean ± SEM determined by a Mann-Whitney *U* test). **(c) ***Left*: Quantification of αSMA staining. HFF1 fibroblasts were kept at the above indicated concentrations of recombinant OPN (Sigma-Aldrich, SRP3131) for 72 h. Results are given as mean ± SEM determined by One-Way ANOVA with Tukey post test. *Right*: Representative immunofluorescence images for αSMA of OPN-treated HFF1 fibroblasts. The nuclei were stained blue (DAPI). The scale bar equals 20 μm. **(d)** Expression of OPN mRNA (qPCR) normalized to HPRT (Hypoxanthine-guanine phosphoribosyltransferase) as housekeeping gene in HFF1 fibroblasts under control conditions and after OPN-knockdown with 3 different siRNAs, (*n* = 3 per group; results are given as mean ± SEM). **(e)** Expression of ACTA2 (αSMA), COL1A1 (collagen 1) and FAP (fibroblast activation protein alpha) mRNA (qPCR) normalized to HPRT in HFF1 fibroblasts 24 h after OPN knockdown (*n* = 3 per group; results are given as mean ± SEM determined by a Mann-Whitney U test for each gene). **(f)** Quantification and representative western blots for αSMA and Collagen 1 protein abundance in RIM fibroblasts ± OPN knockdown, normalized to GAPDH, (*n* = 5 per group; results are given as mean ± SEM determined by a Welch t test).

Based on the RNA-seq data, we went on to characterize the function of OPN in RIM, as exaggerated OPN expression has been linked to systemic sclerosis and other fibrotic diseases^33,35^. Both OPN mRNA and protein expression were significantly higher in RIM compared to control fibroblasts (*p* = 0.0310 and *p* = 0.0079, Fig. 3a and b). In order to investigate whether OPN acts as either a disease driver or a potential biomarker in RIM, we exposed dermal HFF1 fibroblasts to recombinant OPN in ascending concentrations and performed an OPN knockdown in HFF1 and RIM fibroblasts in parallel. Although, we found a concentration-dependent increase in fibrillary αSMA expression in reaction to recombinant OPN, (Fig. 3c), the knockdown of the endogenous OPN expression (Fig. 3d) did not affect the cellular myofibroblasts phenotype. In HFF1, mRNA expression of *ACTA2*, *COL1A1* and *FAP* (fibroblast activation protein alpha) remained unaltered (Fig. 3e). In RIM fibroblasts, protein expression of αSMA and Collagen 1 showed similar results (Fig. 3f), indicating that the profibrotic phenotype is not revertible by downregulation of OPN.

### Characterization of the regulatory effects of β-catenin and myc signaling on the myofibroblasts phenotype in RIM

**Fig. 4:**
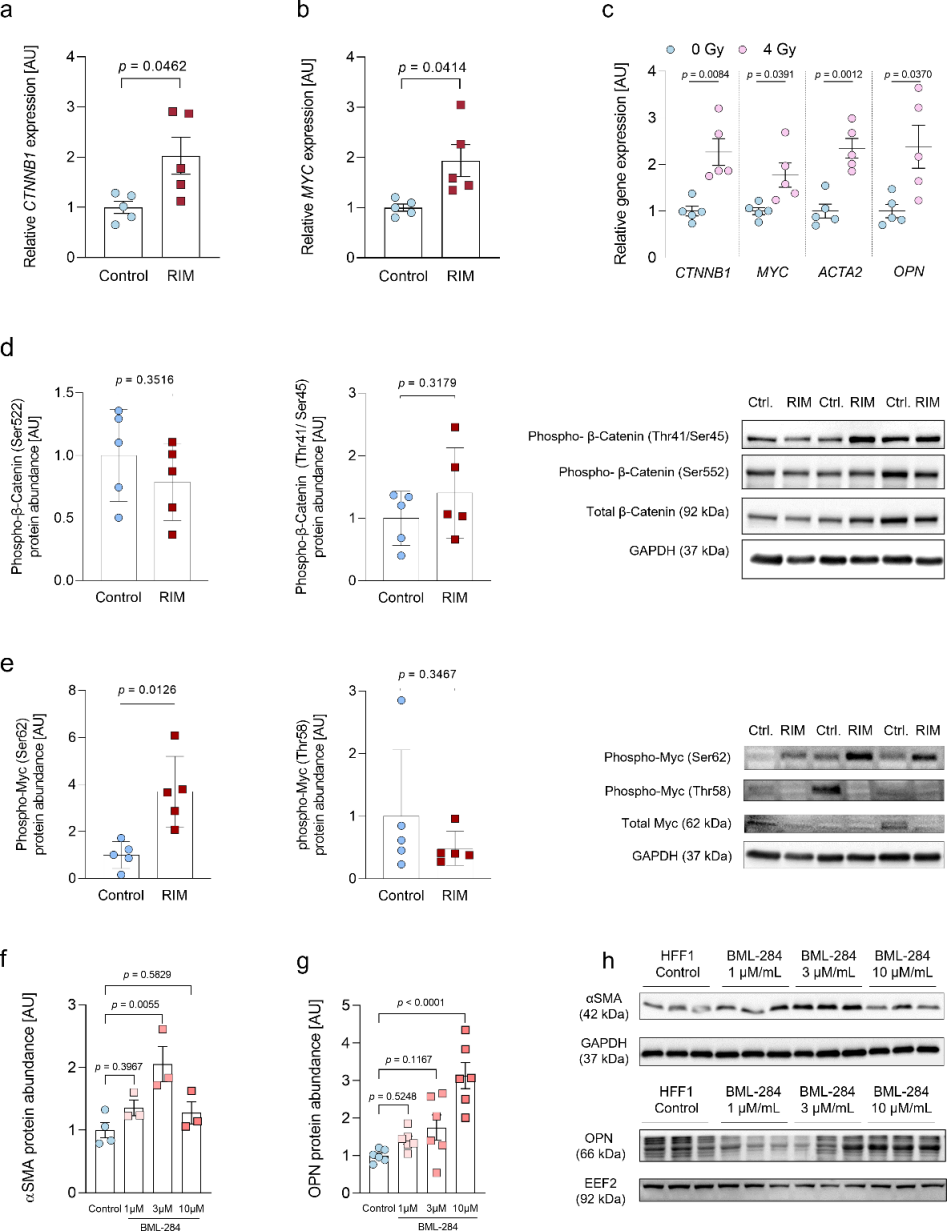
Characterization of the regulatory effects of β-Catenin and Myc signaling on the myofibroblasts phenotype in RIM**. (a)** Expression of *CTNNB1* (β-Catenin) mRNA (qPCR) normalized to *EEF2* as housekeeping gene in control and RIM-fibroblasts, (*n* = 5 per group; results are given as mean ± SEM determined by a Welch *t* test). **(b)** Expression of *Myc* mRNA (qPCR) normalized to *EEF2* in control and RIM-fibroblasts, (*n* = 5 per group; results are given as mean ± SEM determined by a Welch *t* test). **(c)** Radiation-dependent expression of *CTNNB1*, *Myc*, *ACTA2* (αSMA) and *OPN* mRNA (qPCR) normalized to *EEF2* in control fibroblasts after 24 h of exposure to 4 Gy of gamma irradiation, (*n* = 5 per group; results are given as mean ± SEM determined by a Welch *t* test for each gene). **(d)** Quantification and representative western blots of β-Catenin phosphorylation at Ser552 and Thr41/Ser45 in control and RIM fibroblast normalized to GAPDH, (*n* = 5 per group; results are given as mean ± SEM determined by a Welch t test). **(e)** Quantification and representative western blots of Myc phosphorylation at Ser62 and Thr58 in control and RIM fibroblast normalized to GAPDH, (*n* = 5 per group; results are given as mean ± SEM determined by a Welch t test). **(f)** Quantification of αSMA protein abundance in HHF1 fibroblasts upon pharmacological activation of Myc signaling with 1, 3 and 10 µM BML-284 or vehicle control (1 µL DMSO/mL medium), normalized to GAPDH, (n_control_ = 4, n_BML-284_ = 3 per group; results are given as mean ± SEM determined by a One Way ANOVA and Dunnett’s multiple comparison test). **(g)** Quantification of OPN protein abundance in HHF1 fibroblasts upon pharmacological activation of Myc signaling with 1, 3 and 10 µM BML-284 or vehicle control (1 µL DMSO/mL medium), normalized to GAPDH, (*n* = 6 per group; results are given as mean ± SEM determined by a One Way ANOVA and Dunnett’s multiple comparison test). **(h)** Representative western blots for f and g.

Since aberrant β-Catenin and Myc signaling have been shown to exert profibrotic effects in systemic sclerosis, pulmonary and renal fibrosis and induce OPN expression^37,40–45^, we performed qPCR to confirm the RNA-Seq results and found that *β-Catenin* and *Myc* mRNA were significantly higher expressed in RIM compared to control (*p* = 0.0462 and *p* = 0.0414; Fig. 4a and b). Further histological analysis on available samples from two RIM patients demonstrated expression of αSMA, β-Catenin, Myc and OPN (Figure S2). To establish a link between irradiation and the identified fibroblast phenotype in RIM, control fibroblasts were exposed to a single dose of 4 Gy X-irradiation and subsequently analyzed after 24 h. As determined by qPCR, we found a distinct increase in *CTNNB1*, *MYC*, *ACTA2* and *OPN* mRNA expression induced by X-irradiation (Fig. 4c). Since, the biological activity of β-Catenin and Myc is linked to their respective phosphorylation status^46^, we performed phosphorylation analysis by western blot. With regard to β-Catenin phosphorylation, no significant differences were detectable between control and RIM fibroblasts (Fig. 4d). For Myc activity, two phosphorylation sites are considered particularly important. Phosphorylation at serine 62 has been linked to increased Myc activity, whereas phosphorylation at threonine 58 leads to Myc degradation and thereby to decreased activity^46^. We found a 3.7-fold higher Myc phosphorylation at serine 62 in RIM fibroblasts compared to control (*p* = 0.0126), while there was no significant difference in threonine 58 phosphorylation (Fig. 4e). Therefore, subsequent experiments focused on the effects of pharmacological Myc modulation on the myofibroblast phenotype in RIM. First, we sought to answer the question whether pharmacological activation of Myc signaling (BML-284 ^47^; 1, 3 and 10 µM for 8 h) was sufficient to induce a myofibroblast phenotype in HFF1 fibroblasts. Indeed, both αSMA and OPN protein expression increased in a concentration-dependent manner after 8 h of pharmacological treatment with BML-284 (Fig. 4f).

### Mesalazine modulates the cellular localization and phosphorylation status of myc

**Fig. 5:**
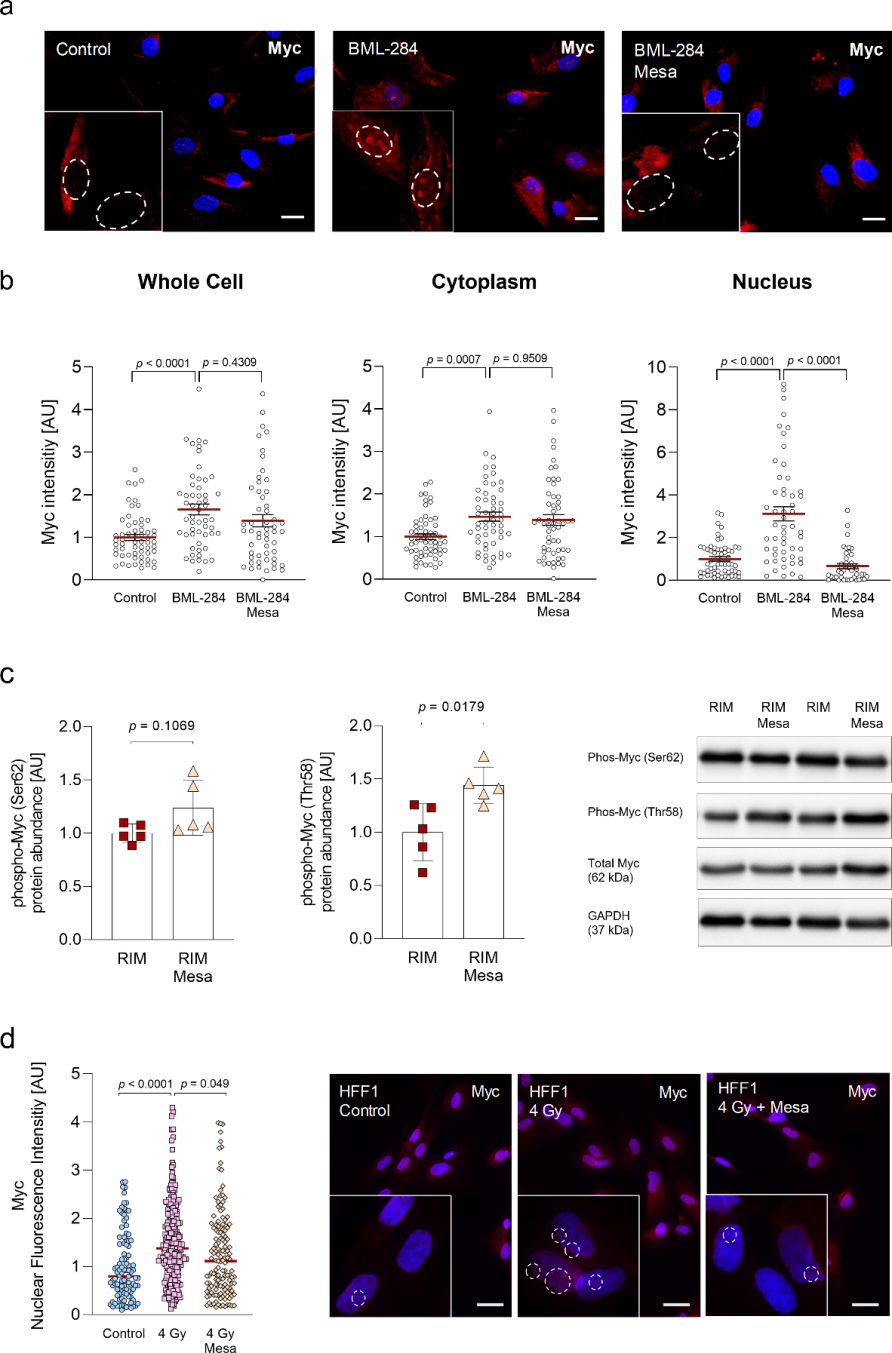
Impact of mesalazine treatment on the cellular localization and phosphorylation status of Myc.** (a)** Representative immunofluorescence images of Myc (red) in human skin fibroblasts (HFF1) upon treatment with vehicle control (1 µL DMSO/mL medium); 1 µM BML-284 or 10 mM mesalazine subsequent to 1 µM BML-284. The nuclei were stained with DAPI (blue). The scale bars equal 20 μm. **(b)** Quantification of whole cell, cytoplasmic and nuclear Myc fluorescence intensity relative to the total cellular area determined using the CellProfiler software (version 4.2.1); (Whole cell [*n* = 66, 108, 62], Cytoplasma [*n* = 65, 110, 62], Nucleus [*n* = 67, 114, 63], results are given as mean ± SEM determined by a Brown-Forsythe and Welch ANOVA test). **(c)** Quantification and representative western blots of Myc phosphorylation at Ser62 and Thr58 normalized to GAPDH in RIM fibroblast under basal conditions and after 10 mM mesalazine treatment for 24 h, (*n* = 5 per group; results are given as mean ± SEM determined by a Welch t test). **(d)** Quantification and representative images of nuclear Myc fluorescence intensity relative to the total cellular area in HFF1 fibroblasts under control conditions, 4 Gy x-irradiation ± 10 mM mesalazine treatment for 24 h [*n* = 103, 251, 141]. The nuclei were stained with DAPI (blue). The scale bars equal 20 μm. Results are given as mean ± SEM determined by a Brown-Forsythe and Welch ANOVA test).

As we found increased Myc activity in RIM fibroblasts and pharmacological experiments indicated a positive correlation of Myc activation and the induction of myofibroblast differentiation (Fig. 4f-h), our aim was the modulation of this signaling pathway with an already clinically approved compound. Mesalazine is used for the treatment of inflammatory bowel disease and has recently been shown to exert antifibrotic effects in vitro and in vivo^22–24,19^. The exact mechanism of action cannot be attributed to the modulation of a single pathway^20^. Inhibition of NFκB, activation of PPARγ, inhibition of TGFβ-SMAD-signaling but also inhibition of Myc signaling have been reported^24,48^. First, we examined the effects of mesalazine on the cellular localization of Myc. Immunofluorescence analysis of HFF1 fibroblasts showed that Myc was mainly located in the cytoplasm, whereas pharmacological treatment with BML-284 resulted in an overall increased Myc fluorescence signal intensity and significantly increased nuclear localization (*p* = 0.0007; Fig. 5a left and middle image). Notably, co-treatment with mesalazine reduced overall Myc fluorescence intensity and abolished the nuclear Myc signal (Fig. 5a middle and left image, b). Probing for a potential mechanism behind these findings, we examined the Myc phosphorylation status after mesalazine treatment in RIM fibroblasts. After 24 h of mesalazine treatment, Ser62 phosphorylation remained unchanged but there was a significant increase in Thr58 phosphorylation (Fig. 5c), which has been linked to Myc degradation^46^. In order to establish a causal relation between radiation and Myc localization, we exposed HFF1 fibroblasts to a single dose of 4 Gy X-irradiation ± mesalazine treatment and analyzed nuclear Myc expression after 24 h via immunofluorescence (Fig. 5d). Radiation significantly (*p* < 0.0001) increased the nuclear Myc signal compared to non-irradiated HFF1 control fibroblasts. In line with the previous results, mesalazine treatment led to a significant (*p* = 0.049) reduction of the nuclear Myc signal.

### Mesalazine reverses the RIM phenotype in vitro and in a patient

**Fig. 6:**
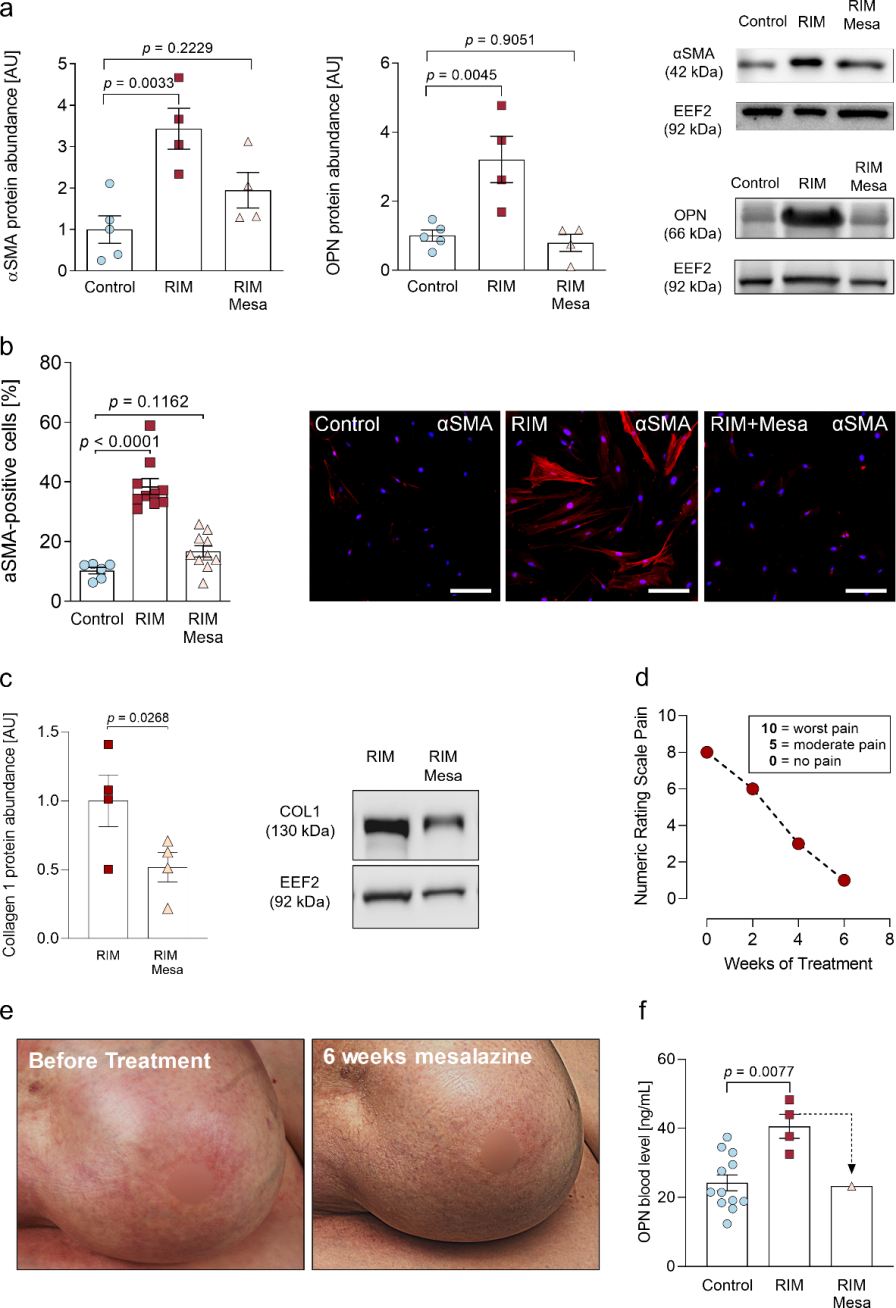
Mesalazine treatment improves the cellular and clinical disease phenotype of RIM**. (a)** Quantification of αSMA (left panel) and OPN (middle panel) protein abundance normalized to EEF2 in primary patient-derived control and RIM fibroblast under basal conditions and after 10 mM mesalazine treatment for 72 h, (control [*n* = 5], RIM [*n* = 4], RIM + Mesa [*n* = 4]; results are given as mean ± SEM determined by a Kruskal-Wallis test with Dunn’s multiple comparisons test). Representative western blots are depicted in the right panel. **(b)** Quantification and immunofluorescence images of αSMA (red) in primary patient-derived control and RIM fibroblast under basal conditions and after 10 mM mesalazine treatment for 72 h, the nuclei were stained with DAPI (blue), the scale bars equal 50 μm, (control [*n* = 6 from *N* = 3 patients], RIM [*n* = 10 from *N* = 5 patients], RIM + Mesa [*n* = 10 from *N* = 5 patients]; results are given as mean ± SEM determined by a One Way ANOVA and Dunnett’s multiple comparison test). **(c)** Quantification and representative western blots of Collagen 1 normalized to EEF2 in RIM fibroblast under basal conditions and after 10 mM mesalazine treatment for 72 h, (*n* = 4 per group; results are given as mean ± SEM determined by a Welch t test).**d)** Graphic representation of the patient’s subjective pain level using the numeric rating scale. **e)** Representative images of the patient before and after oral mesalazine treatment (1 g twice daily) for 6 weeks. *Left image*: Retraction of the right breast with inflammatory erythema, edema, tissue atrophy and marked shiny lesions. The tissue was hardened upon palpatory examination. *Right image*: Erythema and edema were markedly reduced. The tissue was distinctly softer upon palpatory examination and shiny lesions were reduced. **f)** ELISA measurement of OPN concentration in the peripheral blood of healthy control patients and patients with RIM. One RIM patient received oral mesalazine treatment (1 g twice daily) for 6 weeks, (control [*n* = 12], RIM [*n* = 4], RIM + Mesa [*n* = 1]; results are given as mean ± SEM determined by a Mann-Whitney *U* test).

We went on to evaluate the mesalazine effects on the myofibroblast phenotype *in vitro.*As antifibrotic effects of mesalazine have been described previously^19,22–24^, we investigated αSMA and OPN protein expression by western blot in primary control, RIM and mesalazine-treated RIM-fibroblasts. After 72 h, no significant difference in αSMA protein expression was found between control and mesalazine-treated RIM-fibroblasts, whereas αSMA expression was significantly elevated in untreated RIM-fibroblasts compared to control (*p* = 0.0033; Fig. 6a left panel). A similar effect was observed for OPN protein abundance (Fig. 6a middle panel). Concomitantly, the exaggerated expression of fibrillary αSMA bundles in RIM-fibroblasts, as determined by immunocytochemical staining, was reduced to control levels after 72 h mesalazine treatment (Fig. 6b). Additionally, we performed a western blot for collagen 1 protein expression in RIM fibroblasts at baseline conditions and after mesalazine treatment. Collagen 1 expression was significantly reduced in mesalazine-treated RIM fibroblasts as compared to untreated controls (*p* = 0.0268, Fig. 6c).

Encouraged by these findings, we conducted an individual treatment attempt in one of the patients whose fibroblasts were characterized in the present study, as previous therapies had failed. We present the case of a 58-year-old Caucasian woman (BMI 34.9 kg/m^2^, non-smoker), who suffered from infiltrating ductal carcinoma of the right breast two years prior to RIM onset. After completion of chemotherapy (paclitaxel) and radiation therapy for 3 months, the patient was treated with exemestane. A complete list of the patient’s medication can be found in Table 1. 5 months after completion of radiation therapy, she first noticed erythema, severe pain, edema, tissue induration and a rapid shrinking of the right breast. The changes in skin condition were confined to the radiation port. The diagnosis of RIM was made based on the clinical presentation and histological analysis. Initial treatment consisted of topical and systemic steroids, methotrexate, physical therapy and UVA1 treatment without significant clinical response. The patient was informed about the off-label use of mesalazine and gave written informed consent to be treated with orally administered mesalazine (1000 mg, twice daily) for 6 weeks. Telephone interviews to determine pain and tolerability were conducted at weeks 2 and 4. After 6 weeks, a clinical examination was performed. Systemic mesalazine treatment led to a striking improvement in pain (reduction from 8 to 2 NRS pain, Fig. 6d), tissue softening upon palpatory examination and pronounced reduction of erythema and edema (Fig. 6e). We applied a modified version of the LoSCAT (Localized Scleroderma Cutaneous Assessment Tool)^49–51^ to quantify the skin changes during treatment in the single affected region. The modified LoSCAT can be found in Table S2. Briefly, the patient achieved an 80% reduction of disease activity and a modest reduction of disease damage during the mesalazine treatment. No serious adverse events were reported and laboratory results indicated good tolerability. OPN plasma levels were determined by ELISA in blood samples that were obtained for routine monitoring of liver and kidney function as well as blood count. Compared to healthy controls (24.23 ± 2.3 ng/mL), RIM patients had significantly elevated plasma OPN (*p* = 0.0077; 40.61 ± 3.5 ng/mL). After 6 weeks of mesalazine treatment, plasma OPN was reduced to average control levels in our patient (Fig. 6f). Medication was stopped later by the patient because of stable disease.

**Table 1:** Medication of the study patient.

Drug	Indication
Budesonide/ Formoterol 320/9 µg inhaler	Asthma
Salbutamol 0.1 µg	Asthma
Candesartan 8 mg	Arterial hypertension
Levothyroxine 175 µg	Hypothyroidism
Apixaban 5 mg	Venous thromboembolism prophylaxis
Exemestane 25 mg	Anti-estrogen therapy
Alendronate 70 mg	Osteoporosis
Vitamin D 20.000 IE	Osteoporosis

## Discussion

In this study, we present the first functional and molecular characterization of patient-derived RIM-fibroblasts. We demonstrate a pronounced myofibroblast phenotype in RIM accompanied by increased Myc phosphorylation and enhanced OPN expression, which is considered a factor contributing to the evolution of fibrosis as well as a potential fibrosis biomarker^23,34,35,52^. Based on our pre-clinical findings, we provide a translational proof-of-concept for a successful therapy with repurposed mesalazine to improve the RIM phenotype. Although heterogeneous at first glance^53^, fibroproliferative diseases share various motifs on the cellular and molecular level^16^. Compared to RIM, systemic sclerosis (SSC) is a somewhat analogous fibroproliferative disease in which remodeling of the skin, but also of the internal organs, occurs^54^. Based on the clinical resemblances between SSC and RIM, the existence of shared molecular and cellular features is conceivable. Myofibroblast differentiation and subsequent ECM accumulation are the prerequisites for fibrotic remodeling irrespective of the affected organ^8,16^. At baseline, RIM-fibroblasts demonstrated strong expression of αSMA (Fig. 1a and b). In line with their differentiated phenotype, RIM-fibroblasts displayed reduced cell proliferation and migration rates (Fig. 1c and d), consistent with previous findings in different fibrotic entities^23,24,34^. Notably, RIM-fibroblasts maintained their phenotype throughout several passages in cell culture. Similar observations have been made in primary fibroblasts isolated of right atrial tissue of patients with permanent atrial fibrillation, which maintained their myofibroblast phenotype after primary and secondary culture durations of up to 35 days^10^. Hence, genetic patterns and epigenetic regulation of the myofibroblast phenotype seem likely. Thus, in order to gain an objective and unbiased insight into gene expression in control and RIM-fibroblasts, we conducted an RNA-Seq, revealing pronounced differential gene expression between the two groups (Fig. 2, Table S1). We found significantly higher OPN mRNA and protein levels in RIM (Table S1, Fig. 3a and b). OPN has been identified as a relevant cytokine in hepatic, cardiac and pulmonary fibrosis as well as in SSC^19,22,33,55^. The role of OPN in myofibroblast differentiation and subsequent fibrosis was further examined in the present study, demonstrating that stimulation of HFF1 fibroblasts with recombinant human OPN led to significant, concentration-dependent αSMA expression (Fig. 3c). Although exogenously applied OPN led to myofibroblast differentiation, the knockdown of endogenous OPN using siRNA, failed to improve the myofibroblast phenotype in HFF1 and primary RIM fibroblasts (Fig. 3). On one hand, these results support the hypothesis that OPN could rather serve as a biomarker of RIM disease activity instead of a disease driver. On the other hand, limiting factors such as the optimal time point of phenotypic analysis after the knockdown, compensatory mechanisms like transcriptional adaptation^56^and the presence of already synthesized OPN within the cultures have to be taken into account as studies on OPN knockout models demonstrated a central role in myofibroblast differentiation and skin fibrosis development^33,57^. Future studies on this matter have to address these open questions, preferably in genetic knockout models.

**Fig. 7:**
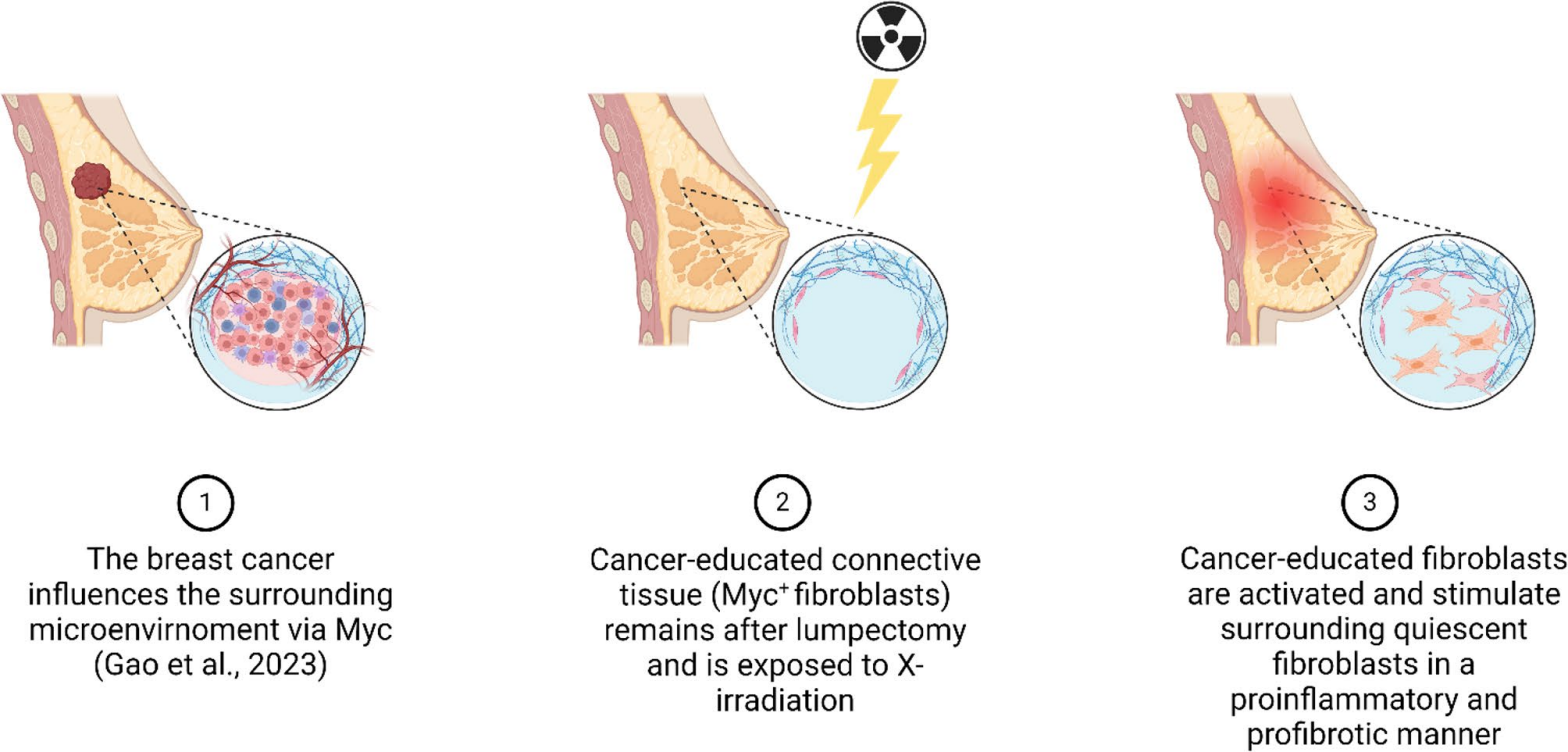
Working hypothesis on RIM pathogenesis. Residual Myc^+^ cancer-educated fibrblasts (CEFs) remain in the affected breast after lumpectomy. Subsequent radiation therapy activates CEFs. Activated CEFs stimulate quiescent tissue fibroblasts and foster fibrosis and inflammation. Created with BioRender.com.

Fibrosis development is a multifactorial process, which is incompletely understood. Relevant inductors of fibrosis, include among others: TGFβ-signaling, hypoxia and oxidative stress^58^. However, as the expression of TGFβ remained unaltered in RIM-fibroblasts compared to control (Table S1) and reactive oxygen species levels were slightly, but not significantly elevated in cultured RIM-fibroblasts (Figure S3), we evaluated mRNA-expression levels of non-canonical regulators of fibrosis^59^and found significantly elevated expression of Myc and one of its upstream regulators, β-Catenin^40,60^. Probing for the initiating stimulus of RIM development, we exposed control fibroblasts to 4 Gy of X-irradiation, as radiation therapy is the prerequisite for RIM development^5^. Indeed, irradiated fibroblasts expressed significantly higher levels of *β-Catenin*, *Myc*, *αSMA* and *OPN* mRNA compared to control (Fig. 4c). However, as determined by phosphorylation-specific western blots, we could only verify increased Myc activation in RIM indicated by enhanced Ser62 phosphorylation (Fig. 4e), whereas there were no differences in β-Catenin phosphorylation (Fig. 4d). Myc has been demonstrated to play a role in DNA double-strand break repair after radiation exposure^61^, which is further supported by the finding of increased nuclear Myc expression in radiation-exposed HFF1 fibroblasts (Fig. 5d). Interestingly, radiation-independent overexpression of Myc has also been found in primary skin fibroblasts derived from SSC patients and has been linked to the myofibroblast phenotype^36,37^. Furthermore, aberrant Myc activation has been shown to induce OPN expression^42^and a general profibrotic role for Myc is further corroborated by a study on renal fibrosis, which demonstrated fibroblast activation by direct binding of Myc to the promoter of integrin αv, leading to alternative activation of TGFβ signaling^44^. It is however currently unclear what factors lead to the permanent maintenance of the dysfunctional fibroblast phenotype in a small cohort of breast cancer patients, whereas the majority of patients receiving radiotherapy do not develop RIM^4^. Although we can only hypothesize at this point, Myc has been reported to mediate a pathological crosstalk between (breast) cancer and the tumor microenvironment (TME), in which fibroblasts are among the most abundant cells^62^. Myc-positive cancers stimulate cancer-associated fibroblasts, which themselves subsequently show Myc activation^62,63^. The tumor microenvironment (TME) is heterogeneous and less well-defined than the tumor itself. This factor may be particularly relevant in obese patients, as there is more adipose and connective tissue surrounding the tumor. Indeed, clinical observations have linked the development of RIM to above-average breast volume^4^. It is therefore conceivable that although the primary cancer has been surgically removed, distant remnants of the TME remain^64^, in which cancer-educated fibroblasts could then be activated in a “second hit” fashion following radiotherapy, leading to further paracrine fibroblast activation and a self-perpetuating loop of inflammatory fibrosis^65^ (Fig. 7). This hypothesis is strengthened by our sequencing data, indicating significant enrichment of Myc-regulated genes and cancer-specific pathways in RIM fibroblasts supporting the notion of “cancer education” in those fibroblasts (Fig. 2d, f) as well as our data on increased Myc expression and nuclear localization after radiation exposure (Figs. 4c and 5d). For future studies on this topic, it will be necessary to use animal models to investigate the interactions of residual TME, Myc and radiation in RIM pathogenesis.

We aimed to establish a therapeutic approach based on our findings. Our previous studies have demonstrated antifibrotic and anti-OPN effects of mesalazine in models of cardiac and cutaneous fibrosis^20,22,23^. In accordance with data on the use of mesalazine in inflammatory bowel diseases^48,66^, we found that mesalazine reduced pharmacologically induced activation of Myc signaling in skin fibroblasts to control levels (Fig. 5a-c) and led to drastically reduced myofibroblast differentiation as well as αSMA, OPN and Collagen 1 protein expression (Fig. 6a-c). Our data indicate that the observed effects could be mediated by induction of Myc degradation^46^, as determined by increased THr58 phosphorylation, which in turn could be responsible for the abolished nuclear Myc translocation. In order to investigate the translational value of these findings, we measured OPN plasma concentrations in healthy subjects and RIM patients via ELISA and found significantly higher OPN plasma concentrations in RIM (Fig. 6f), which has recently been confirmed in systemic sclerosis as well^67^. In an individual treatment attempt, a patient was treated with 2 g of orally administered mesalazine for 6 weeks and experienced pronounced symptomatic relief and good tolerability of the therapy (Fig. 6d-e). Two similar cases have been published in 1982 and 1999 in which patients suffering from generalized morphea (not radiation-induced) were successfully treated with sulfasalazine^68,69^, which is metabolized to mesalazine as active moiety^20^. Furthermore, the OPN plasma concentration was reduced to average control levels after mesalazine treatment (Fig. 6f). OPN has demonstrated promising potential as a novel RIM biomarker, which responded to therapeutic interventions and thus could be adapted as a marker of therapeutic success in the clinical routine. Our findings could be of potential clinical relevance as there is currently no standardized therapy for RIM. Most approaches include broad immunosuppression and thus harbor potentially serious side effects^5^. Mesalazine on the other hand has been approved for several decades and offers good tolerability at low cost of treatment^20^.

### Potential limitations

There are limitations to the current study, which have to be addressed in future research on the subject. As RIM is considered a rare disease^1^, the sample size and availability is very limited. We were able to provide a first functional and molecular characterization of RIM-derived fibroblasts, yet, the underlying mechanisms inducing RIM in a subpopulation of breast cancer patients remain to be clarified. In particular, the exact role of radiation therapy in RIM initiation and maintained Myc pathway activation has to be studied in further detail in future research on this matter. Although mesalazine has been used for several decades, its exact mechanisms of action are still not completely understood, broad inhibition of several fibrotic pathways is likely^20^ and spontaneous symptomatic improvement can only be ruled out by a randomized controlled trial. Furthermore, to determine the exact mode of action of mesalazine in RIM, tissue biopsies of the affected skin before and after mesalazine treatment would be invaluable. However, due to the fibrotic and ulcer-prone nature of the affected skin areas, biopsies should only be performed when necessary and with caution. The unique strength of our study lies in the use of primary patient-derived fibroblasts and blood samples in conjunction with a proof-of-concept clinical treatment attempt. This approach provides new insight into the pathophysiology of RIM offering both scientific and therapeutic avenues in the future.

## Conclusions

The present study is, to the best of our knowledge, the first to provide a characterization of the pathological fibroblast phenotype in RIM. We provide a link between irradiation, myofibroblast differentiation, Myc activation and increased OPN release, thereby presenting novel therapeutic targets and a possible biomarker in this context. Treatment with mesalazine effectively reversed the RIM myofibroblast phenotype potentially via modulation of Myc signaling. Lastly, we provide clinical proof-of-concept for the effectiveness and feasibility of using repurposed mesalazine in RIM. Future clinical and mechanistic studies need to evaluate the role of TME and cancer-associated fibroblasts in RIM, and whether repurposing mesalazine or its derivatives could be an effective pharmacotherapeutic approach to halt or even reverse severe fibrosis and inflammation in RIM.

## Materials and methods

Sample size was not predetermined by statistical methods due to the exploratory nature of this study and limited sample availability. The experiments were not randomized. Investigators were blinded to sample allocation at outcome assessment, where appropriate.

**Informed consent and human sample acquisition.** The collection and study of human tissue and blood samples were reviewed and approved by the Ethics Committee at Technische Universität Dresden, Dresden, Germany in accordance with the Declaration of Helsinki (reference of the ethics committee at Dresden university hospital: EK31022012). After the patients gave their written informed consent, skin punch biopsies and blood samples were collected by physicians of the Dermatology department at Dresden University Hospital “Carl Gustav Carus”.

**Cell isolation and culture conditions.**Primary human skin fibroblasts from control and RIM patients were isolated via outgrowth-technique^10^. All fibroblasts used in this study were cultured at 37 °C and 5% CO2 in Dulbecco’s modified eagle medium (Life Technologies, Carlsbad, CA, USA) supplemented with 10% fetal calf serum (FCS, Life Technologies, Carlsbad, CA, USA) and 1% penicillin/ streptomycin (Life Technologies, Carlsbad, CA, USA). An established human skin fibroblast cell line (HFF1, ATCC: SCRC-1041) was used as a model for mechanistic studies.

**Functional fibroblast characterization.**Experiments to determine fibroblast proliferation, and myofibroblast differentiation were performed as described previously^10,23,34^. To assess the migratory capacity of control and RIM fibroblasts, a modified wound healing assay was performed (Cell Biolabs, Inc., San Diego, USA, #CBA-120-5)^10^. Dividers were placed into a 24-well plate ensuring a cell-free area in the middle of each well. 2.5*10^4^ cells were seeded on each side of the divider. After overnight culture, the dividers were removed and cells were washed with medium twice. Baseline images of the cell-free area (wound) were obtained. After 24 h of culture, the cells were washed with PBS three times and fixated with 4% formaldehyde for 15 min at RT. The nuclei were stained with DAPI and fluorescence images of each well was acquired in order to count the cells migrated into the wound area.

**SDS-PAGE**,** western blotting and immunodetection**. Protein extraction, SDS-PAGE, western blotting were performed as described previously^22,24,34^. Immunodetection and quantification of protein expression were carried out using a Fusion FX device and the Fusion software (Vilber Lourmat Deutschland GmbH, Eberhardzell, Germany). A list of primary and secondary antibodies is provided in Table 2. Full-length images of the corresponding uncropped blots are available in the online supplementary material for each figure.

**Table 2:** Primary and secondary antibodies.  1, Immunocytochemistry ; 2, western blot: 3, immunohistochemistry.

Primary Antibodies				
Protein	Dilution	Source/ Conjugate	Product-Nr.	Usage
αSMA	1:200	Mouse	A5228	ICC^1^/ WB^2^/ IHC^3^
Myc	1:1000	Rabbit	C3956	WB/ ICC
β-Catenin	1:1000	Rabbit	9562	WB/ ICC
Osteopontin	1:1000	Rabbit	ab8448	WB/ IHC
Eukaryontic elongation factor 2 (EEF2)	1:10.000	Rabbit	ab40812	WB
Glyceraldehyde-3-phosphate dehydrogenase (GAPDH)	1:1000	Mouse	sc-365,062	WB
β-Catenin Antibody Sampler Kit	1:1000	Rabbit	#2951	WB
Myc Family Profiling Antibody Sampler Kit	1:1000	Rabbit	#26,717	WB
Collagen 1	1:1000	Rabbit	ab21286	WB
**Secondary Antibodies**				
Anti-rabbit	1:10.000	Peroxidase	111-035-045	WB
Anti-mouse	1:10.000	Peroxidase	A3682	WB
Alexa fluor 546 (Goat-anti-mouse)	1:400	Streptavidin	Z25004	ICC
Alexa fluor 546 (Goat-anti-rabbit)	1:400	Streptavidin	Z25304	ICC

**Histology and image analysis. **For histological analysis, paraffin embedded skin Sect. (5 μm) from representative control and RIM patients were generated by the department of pathology at Dresden University Hospital in the course of clinical diagnostics. A detailed description of deparaffinization and subsequent immunohistochemical staining was published recently^34^.

Fluorescence images were acquired with a Keyence BZ-X710 All-in-One Fluorescence Microscope (Keyence Corporation of America, Itasca, USA).

**Osteopontin knockdown.** Lipofectamine (Thermo Fisher, Waltham, Massachusetts, USA; #13778030) was used for siRNA transfection according to the manufacturer’s instructions. The OPN knockdown was performed using the siRNAs listed in Table 3 (adapted from Patent No.: EP 2 290 063 A1; Table 3):

**Table 3:** OPN siRNAs.

Name of sequence	Sequence (5’ – 3’)
**siOPN1**	-CCAAGUAAGUCCAACGAAA-
**siOPN2**	-TTGGTTGAATGTGTATCTATTTG-
**siOPN3**	-ACUAAAAGCUUCAGGGUUA-

**Fibroblast irradiation.** Fibroblast irradiation was performed at RT using single doses of 200 kV X-rays filtered with 0.5 mm Cu (Yxlon Y.TU 320; Yxlon, Hamburg, Germany). The dose-rate was approximately 1.3 Gy/min at 20 mA. Applied single doses to primary skin fibroblasts were 4 Gy.

**Myc staining.** HFF1 fibroblasts (0.5 × 10^4^/well) were seeded on glass coverslips in 24-well plates. The next day, the cells were either cultured in drug-free medium (1 µL DMSO/mL vehicle control) or stimulated with BML-284 (Selleck Chemicals GmbH, Germany; S8178 ) (1 µmol/L) for 1 h, followed by drug-free medium or mesalazine (10 mmol/L) for an additional period of 1 h. After fixation with methanol/acetone (1:1) for 20 min at -20 °C, cells were permeabilized using Triton-X 100 (0.5%). Subsequently, immunostaining for Myc was performed and nuclei were stained with DAPI. To evaluate fluorescence intensity and cellular localization of Myc, images were analyzed using CellProfiler™-Software version 4.2.1 (Broad Institute, Cambridge, USA)^70^.

**RNA isolation**,** cDNA synthesis and qPCR.** SYBR green (Bio-Rad Laboratories GmbH, Munich, Germany) real-time PCR was performed to measure gene expression. Specific primers were purchased from Bio-Rad (Bio-Rad Laboratories GmbH, Munich, Germany). Eukaryotic elongation factor 2 (EEF2) was used as a housekeeping gene. Total RNA was isolated using the RNeasy Micro Kit (Qiagen, Venlo, The Netherlands). Subsequent cDNA synthesis was performed with the PeqGold cDNA synthesis kit (Peqlab Biotechnologie GmbH, Erlangen, Germany). All runs were performed in a CFX96 Touch Deep Well Real-Time PCR detection system (Bio-Rad Laboratories GmbH, Munich, Germany). Samples were amplified in duplicates. CFX manager software (Bio-Rad Laboratories GmbH, Munich, Germany) was used for data analysis. Relative gene expression was calculated to housekeeping gene and subsequently normalized to controls.

**Transcriptome analysis and bioinformatic workflow.** For RNA sequencing primary control (*n* = 5 patients) and RIM (*n*= 5 patients) fibroblasts between passages 2 and 10 were grown on T25 culture flasks and harvested at 80–90% of optical confluence. RNA was isolated as described above. The RNA sequencing was carried out by BGI Genomics (Shenzhen, China). Raw reads were inspected using fastqc^71^, trimmed using trimmomatic^72^and aligned using STAR^73^, GRCH37 was used as reference genome. Read counts were extracted from the alignments using the featureCounts method of the subread package^74^, afterwards DESeq2 was applied to identify differentially expressed genes^75^. Only genes with multiple testing adjusted p-values (padj from DESeq2) < 0.05 were considered statistically significant. Clustermaps and volcano plots were generated using the python libraries matplotlib, seaborn, pandas and scipy. Gene sets for data analysis were provided by GSEA^26–28^and The Harmonizome^29^.

**Individual treatment attempt.** The treatment attempt described in this study was performed in accordance with the declaration of Helsinki. The patient gave written informed consent. The treatment, laboratory controls as well as documentation were performed as a part of the clinical routine at the Department of Dermatology at Dresden University Hospital. Blood samples to monitor liver and kidney function, as well as blood count, were taken every two weeks either at the Dermatology department or at the patient’s general practitioner’s office. A complete list of patient’s medication at the point of mesalazine treatment is provided in Table 1.

**Statistical analysis.** All results are presented as mean ± SEM. For statistical analysis and graphic presentation, Graph Pad Prism software v.8 (GraphPad Software, San Diego, USA) was used. All datasets were tested for normality by using the Kolmogorov-Smirnov test. For comparisons between 2 groups only, Student t test with Welch’s correction if appropriate for normally distributed data or Mann-Whitney U test for non-normally distributed data were used. When comparing 3 groups, 1-way ANOVA or Kruskal-Wallis test was performed with Tukey or Dunn post-test, respectively. *p* < 0.05 was considered statistically significant.

## Data availability

All data obtained in this study is depicted within the figures. The mRNA-Seq dataset generated and analysed during the current study is available in the Sequence Read Archive (SRA) repository, [SUB14471666/ PRJNA1115968].

## Electronic supplementary material

Below is the link to the electronic supplementary material.

Supplementary Material 1

Supplementary Material 2

Supplementary Material 3

## Abbreviations

RIM: Radiation-induced morphea
ECM: Extracellular matrix
αSMA: Alpha-smooth-muscle-actin
OPN: Osteopontin
TME: Tumor microenvironment
