# Supplementary material for: Radiation-induced morphea of the breast – characterization and treatment of fibroblast dysfunction with repurposed mesalazine
Source: Sci Rep. 2024 Oct 30;14:26132. doi: 10.1038/s41598-024-74206-w (PMC11525966; doi:10.1038/s41598-024-74206-w)
Supplement: Supplementary file 2 — Supplementary Material 2 [file 41598_2024_74206_MOESM2_ESM.docx]

Supplementary Material

Radiation-induced morphea of the breast – characterization and treatment of fibroblast dysfunction with repurposed mesalazine

Stephan R. Künzel^1,2,3,4^, Erik Klapproth^1^, Nick Zimmermann^3^, Susanne Kämmerer^1^, Mario Schubert^1^, Karolina Künzel^1^, Maximilian Hoffmann^1^, Stephan Drukewitz^5,6^, Anne Vehlow^7,8^, Jiri Eitler^4^, Marieke Arriens^4^, Jessica Thiel^4^, Romy Kronstein-Wiedemann^4^, Maximiliane Tietze^4^, Stefan Beissert^3^, Bertold Renner^2^, Ali El-Armouche^1,2^, Claudia Günther^3^

1 Institute for Pharmacology and Toxicology, Faculty of Medicine Carl Gustav Carus, Technische Universität Dresden, Dresden, Germany

2 Institute for Clinical Pharmacology, Faculty of Medicine Carl Gustav Carus, Technische Universität Dresden, Dresden, Germany

3 Department of Dermatology, University Hospital and Faculty of Medicine Carl Gustav Carus, Technische Universität Dresden, Dresden, Germany

4 Institute of Transfusion Medicine, Faculty of Medicine Carl Gustav Carus, Technische Universität Dresden, Dresden, Germany

5 Institute of Human Genetics, University of Leipzig Medical Center, Leipzig, Germany

6 Core Unit for Molecular Tumor Diagnostics, NCT Dresden and DKFZ, Dresden, Germany

7 OncoRay - National Center for Radiation Research in Oncology, Faculty of Medicine Carl Gustav Carus, Technische Universität Dresden, Dresden, Germany

8 National Center for Tumor Diseases (NCT), Partner Site Dresden, German Cancer Research Center (DKFZ), Heidelberg, Germany

# Supplementary Figures and Tables

## Supplementary Figures


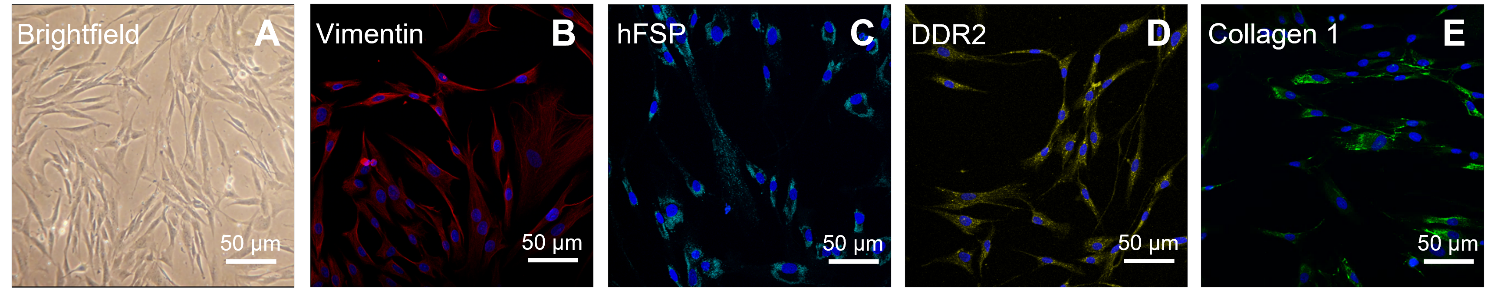


**Figure S1.** **Cytomorphological and immunocytochemical fibroblast characterization.** Representative images of cultured primary human skin fibroblasts isolated by outgrowth technique. The nuclei were stained blue (DAPI). For immunocytochemical staining, Alexa fluor 546 was used. The original channel color (red) was changed to the colors depicted above for better discrimination.

**Figure S2.** **Histological analysis of RIM tissue samples.** Lesional RIM tissue samples from 2 patients were stained immunohistochemically (brown) for αSMA, β-Catenin and Myc. Additionally, immunofluorescence staining for osteopontin (OPN) was performed. OPN was stained red, the nuclei were stained with DAPI (blue), and the scale bars equal 100 µm. The right-hand bar graphs depict the histopathological grading of the respective staining intensity (0 = no expression, 1 = low expression, 2 = moderate expression, 3 = strong expression).


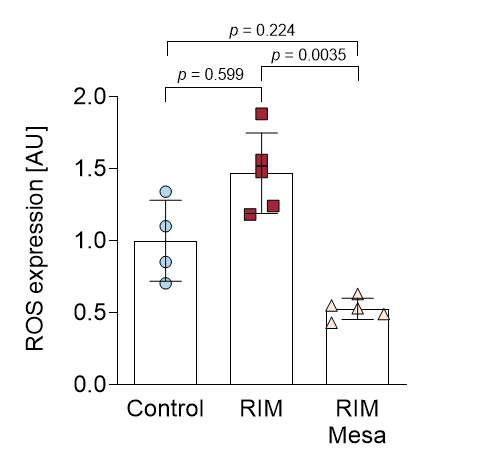


**Figure S3.** **Quantification of reactive oxygen species (ROS) production in control and RIM-fibroblasts using Dihydrorhodamine 123.** RIM-fibroblasts were treated with 10 mmol/L Mesalazine for 1 h before measurement. n_control_ = 4, n_RIM_ = 5, n_RIM+Mesa_ = 5. Results are given as mean±SEM determined by a Kruskal-Wallis test with Dunn’s multiple comparisons test.

## Supplementary Tables

**Table S2. Modified LoSCAT**

| **mLoSCAT** modified Localized Scleroderma Cutaneous Assessment Tool | **LoSAI**  (Localized Scleroderma Skin Activity Index) | | | **LoSDI**  (Localized Scleroderma Skin Damage Index) | | | |
| --- | --- | --- | --- | --- | --- | --- | --- |
|  | **New/ Enlarged**  0 = none  3 = N/ E | **Erythema** 0 = none  1 = pink 2 = red 3 = dark red | **Induration** (skin swelling at edge) 0 = none  1 = mild 2 = moderate 3 = marked | **Dermal atrophy** 0 = none  1 = shiny 2 = visible vessels 3 = cliff drop | **Sub Q/ Deep atrophy** 0 = none  1 = flat 2 = concave 3 = marked | **Dyspigmentation** 0 = none  1 = mild 2 = moderate 3 = marked | **Skin Thickness** (at center) 0 = none  1 = mild 2 = moderate 3 = marked |
| **Right Breast BEFORE Mesa** | 0 | 2 | 3 | 1 | 0 | 0 | 3 |
| **Right Breast AFTER Mesa** | 0 | 0 | 1 | 0 | 0 | 0 | 3 |
| **LoSAI BEFORE MESA** | 5 |  |  | **LoSAI AFTER MESA** | 1 |  |  |
| **LoSDI BEFORE MESA** | 4 |  |  | **LoSDI AFTER MESA** | 3 |  |  |
|  |  |  |  |  |  |  |  |
|  |  |  |  |  |  |  |  |
| **PGA-A^1^ BEFORE Mesa** | 100 |  |  | **PGA-A BEFORE Mesa** | 20 |  |  |
| **PGA-D^2^ AFTER Mesa** | 80 |  |  | **PGA-D AFTER Mesa** | 60 |  |  |
|  |  |  |  |  |  |  |  |
| *^1^ Physician Global Assessment of Disease Acitivity; ^2^ Physician Global Assessment of Disease Damage* | | | | | | | |
