# Supplementary material for: Radiation-induced morphea of the breast – characterization and treatment of fibroblast dysfunction with repurposed mesalazine
Source: Sci Rep. 2024 Oct 30;14:26132. doi: 10.1038/s41598-024-74206-w (PMC11525966; doi:10.1038/s41598-024-74206-w)
Supplement: Supplementary file 3 — Supplementary Material 3 [file 41598_2024_74206_MOESM3_ESM.pdf]

Original Westernblots for Figure 1 B

αSMA
(42 kDa)

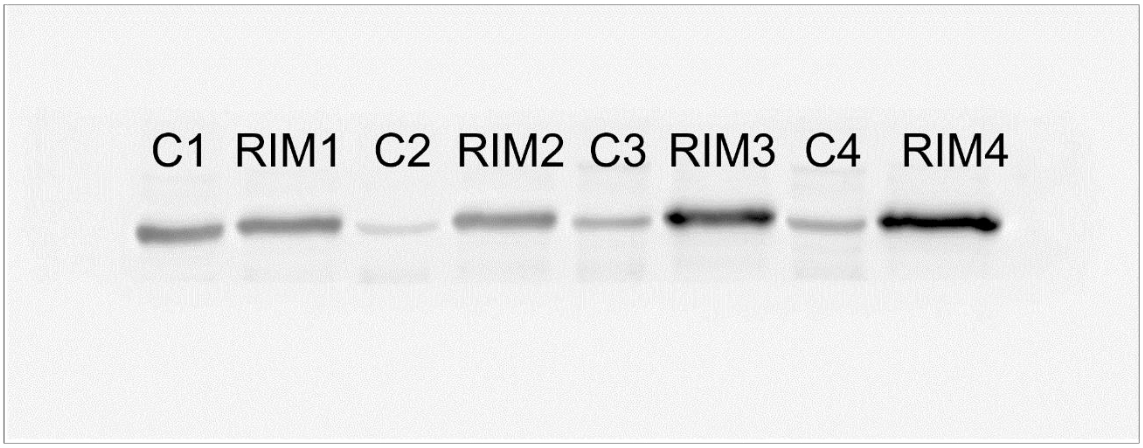

GAPDH
(37 kDa)

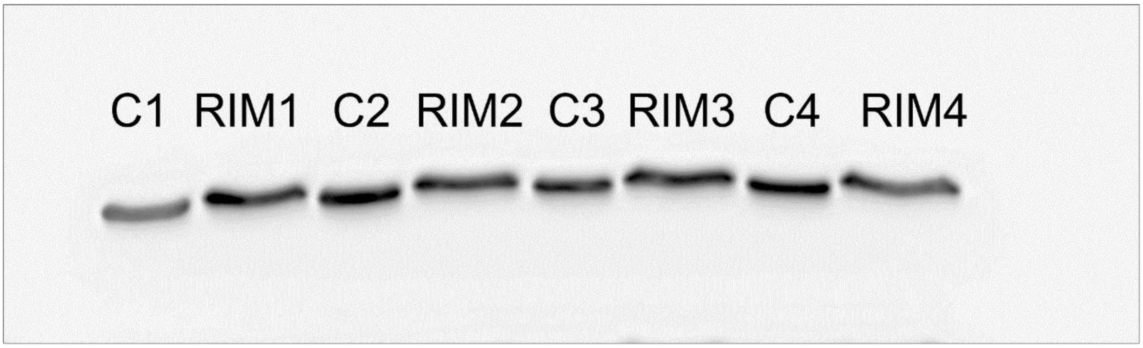

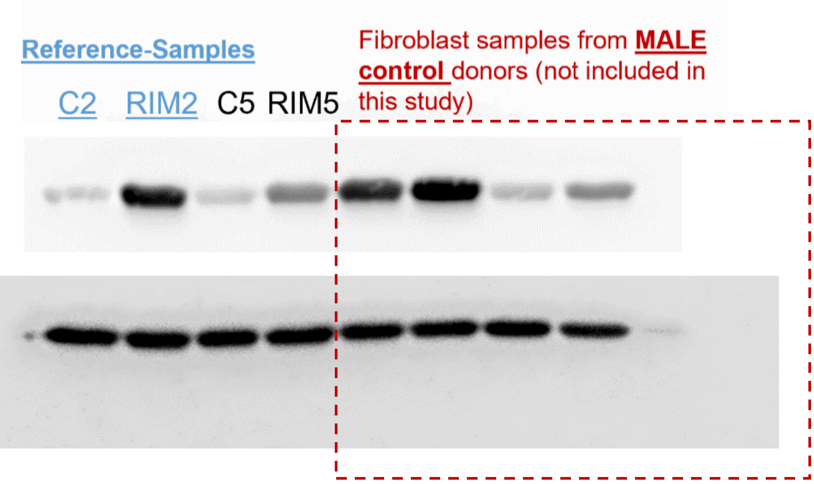

Original Western Blots for Figure 3 b

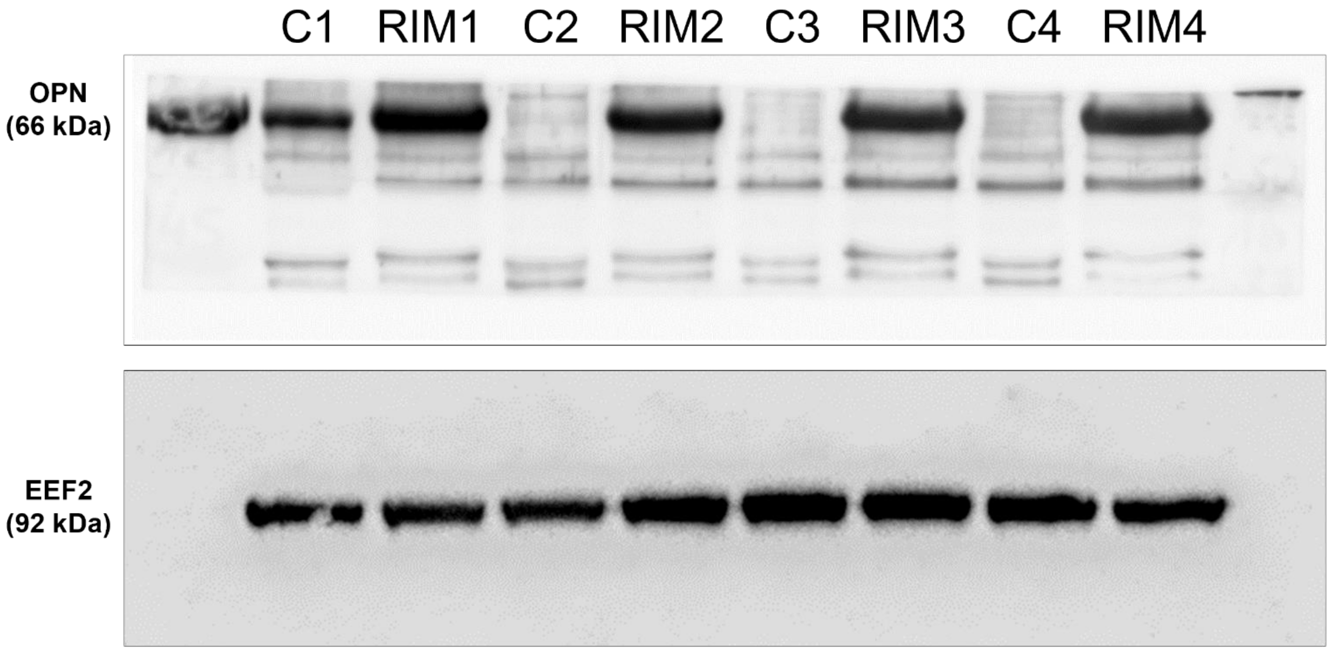

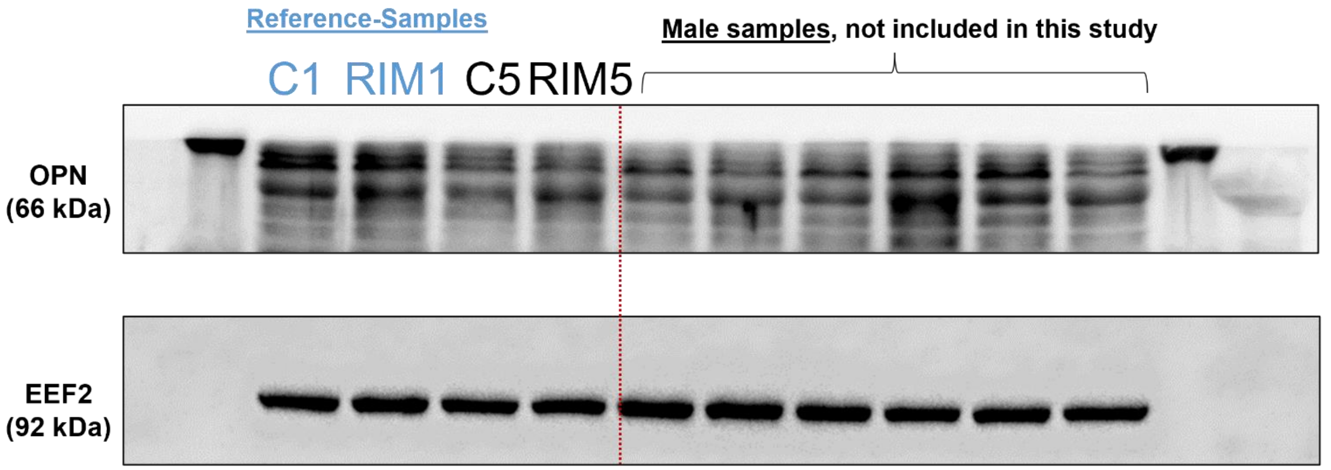

Original Western Blots for Figure 3 f

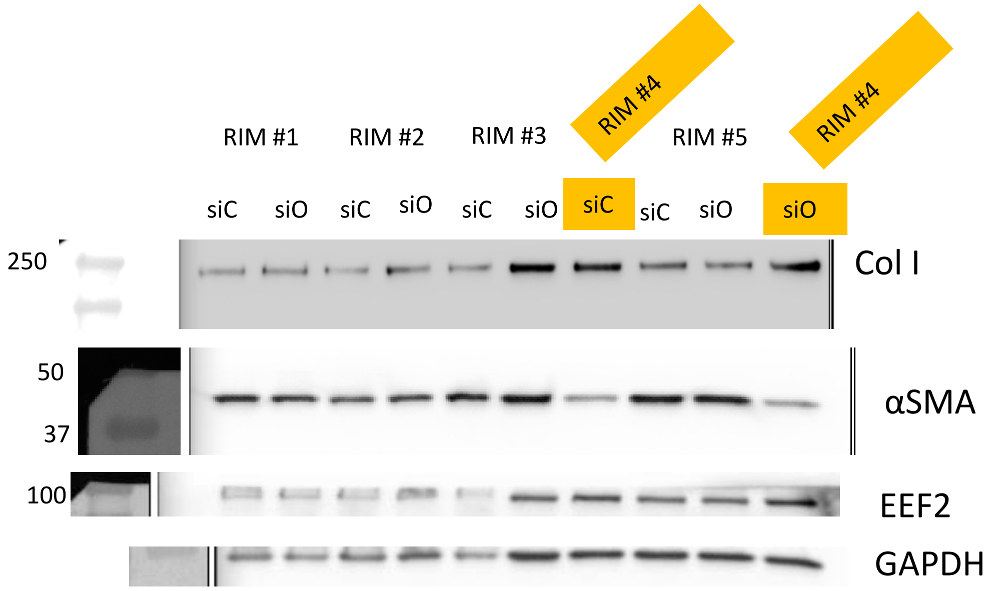

Original Western Blots for Figure 4 d

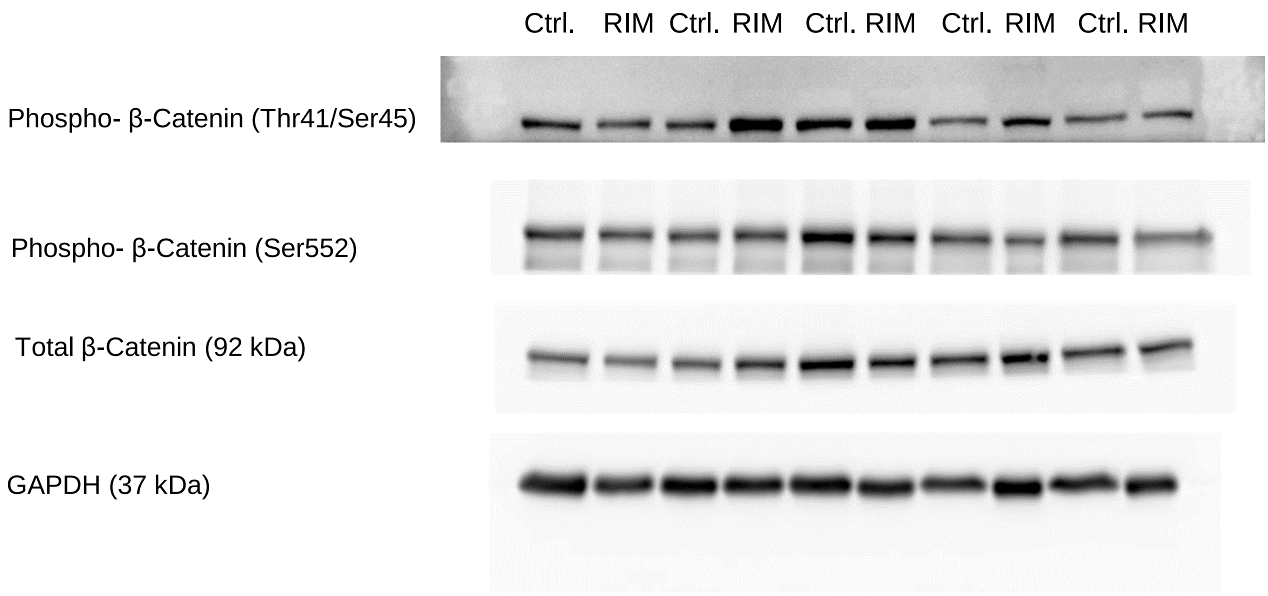

Original Western Blots for Figure 4 e

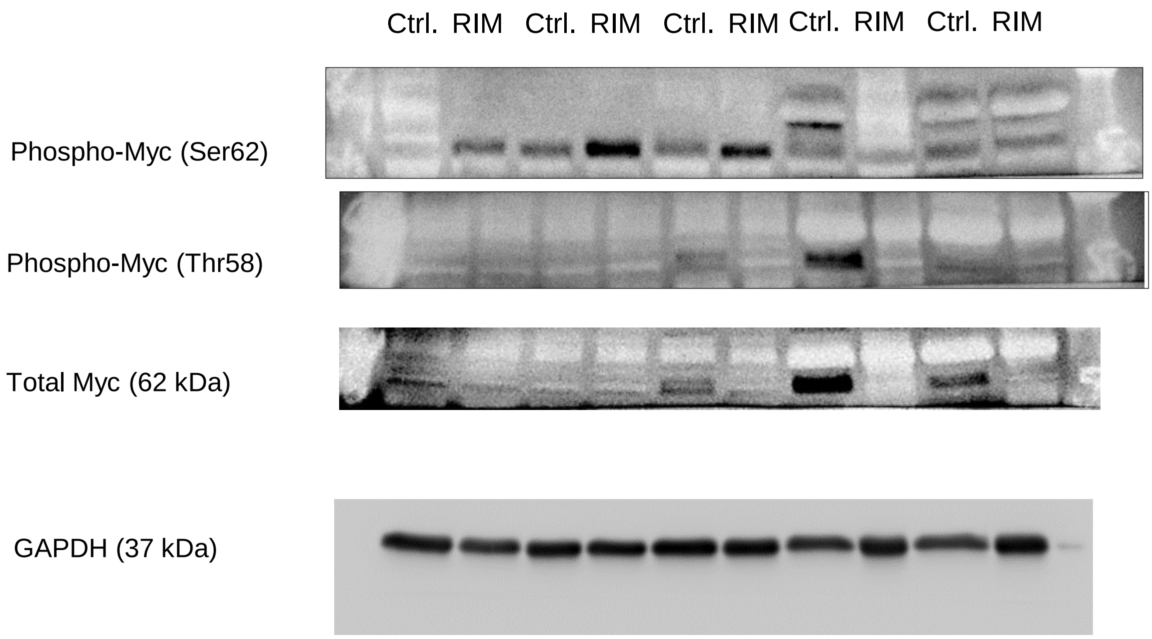

Original Western Blots for Figure 4 h

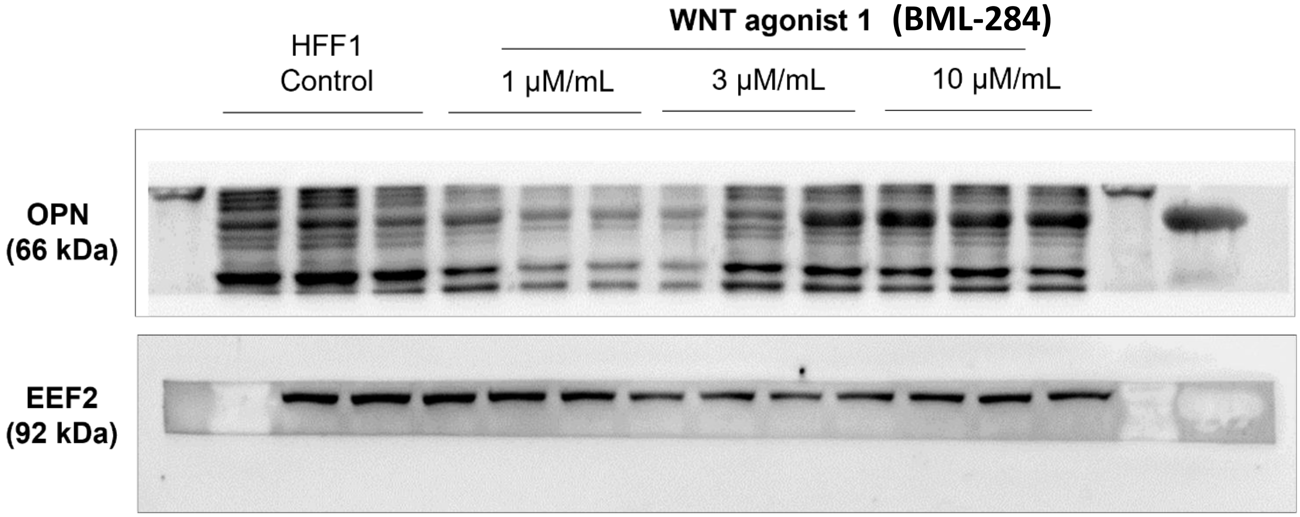

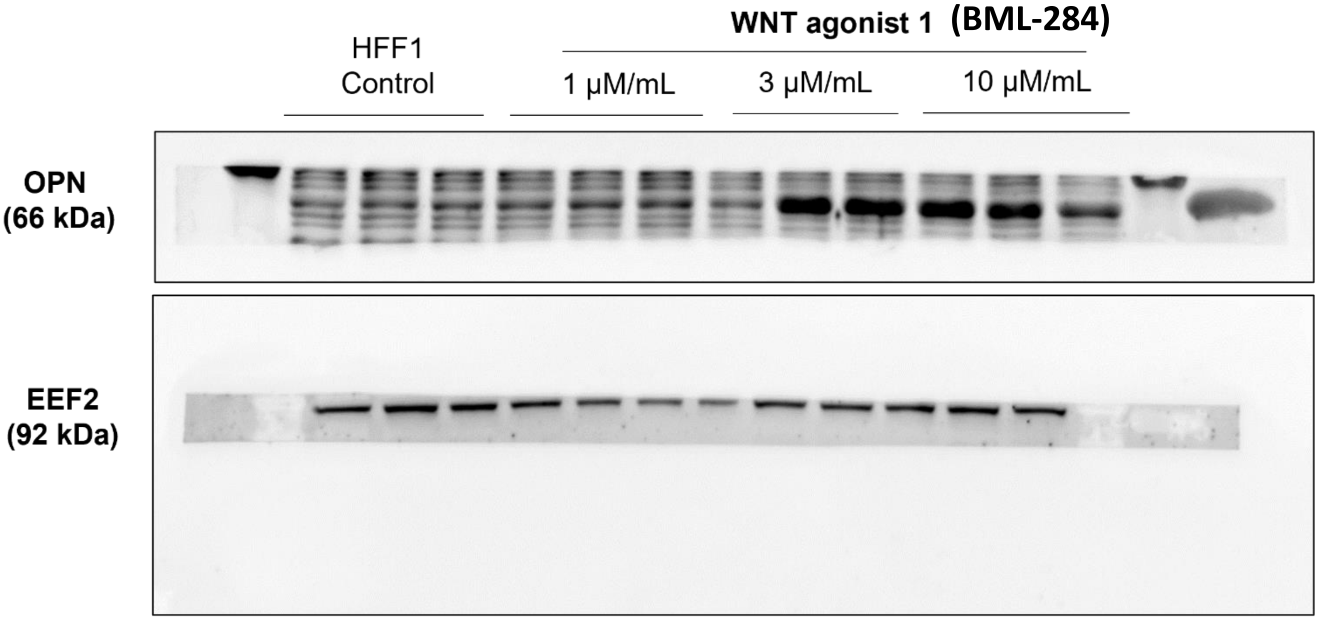

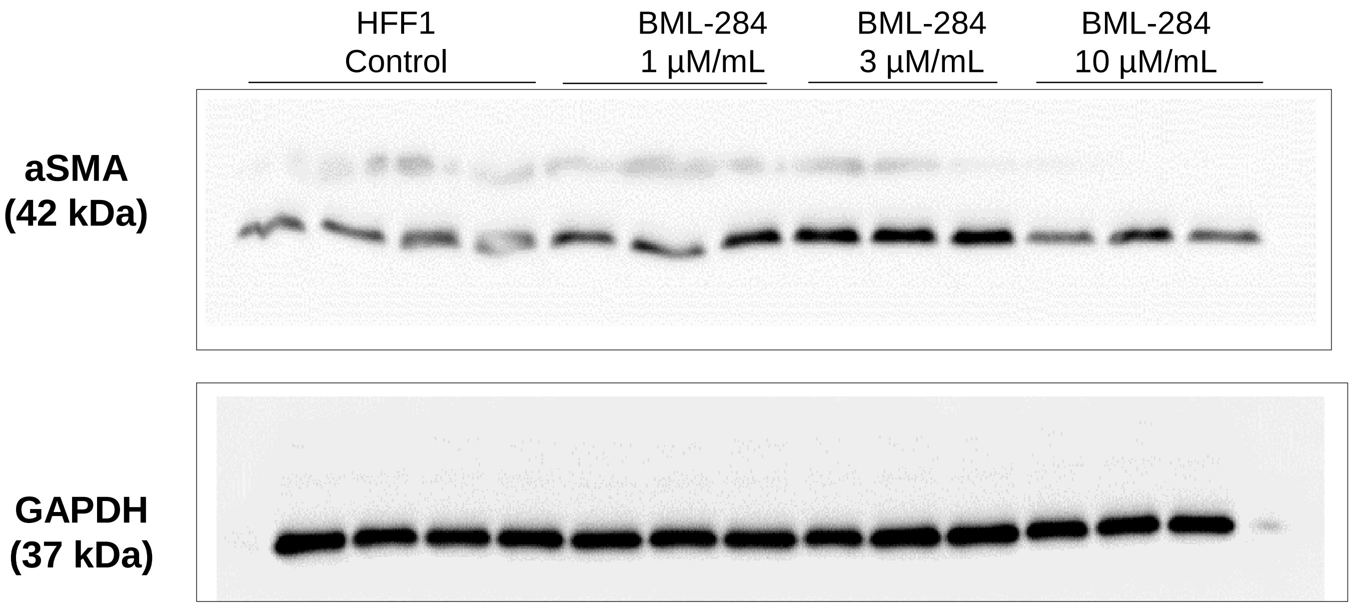

Original Western Blots for Figure 5 c

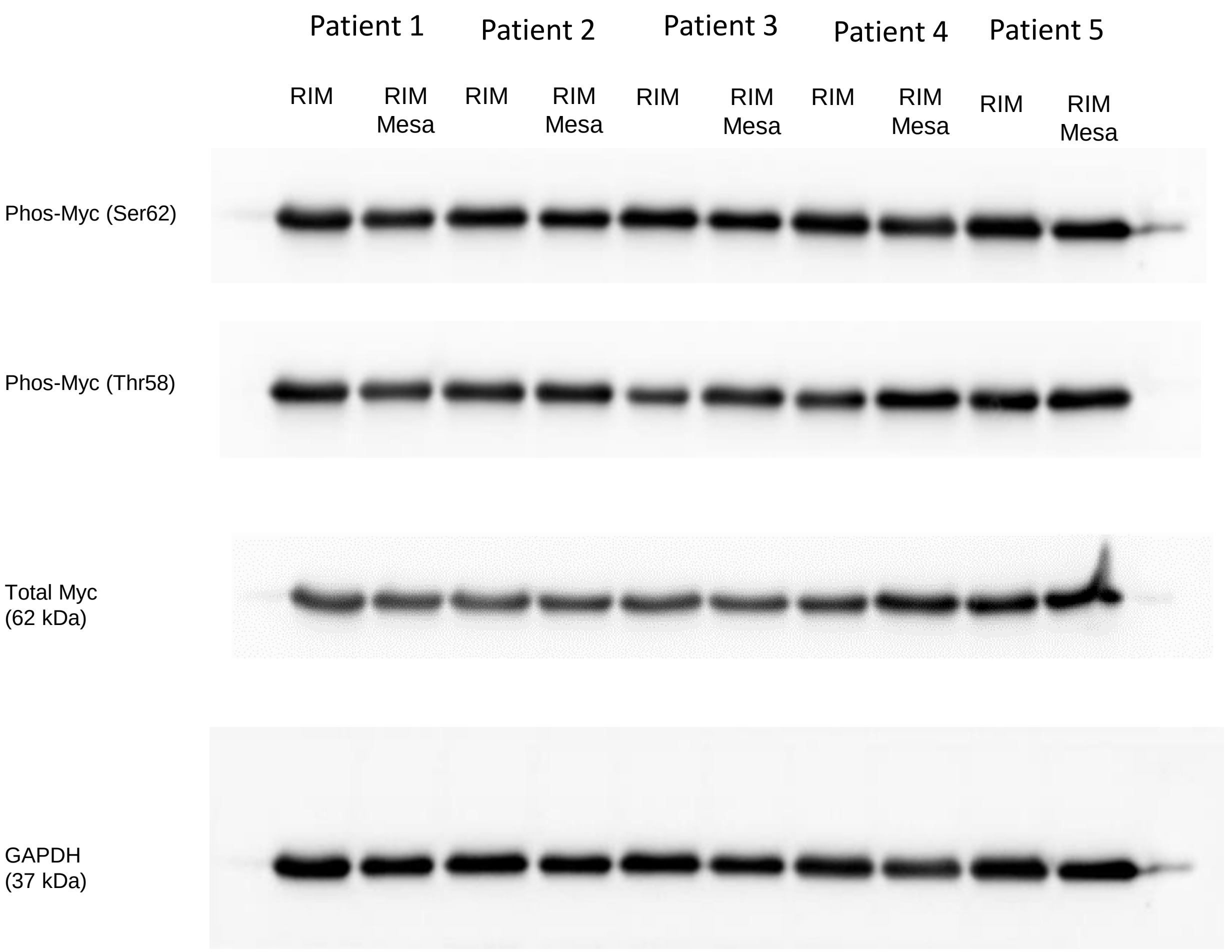

Original Western Blots for Figure 6 a

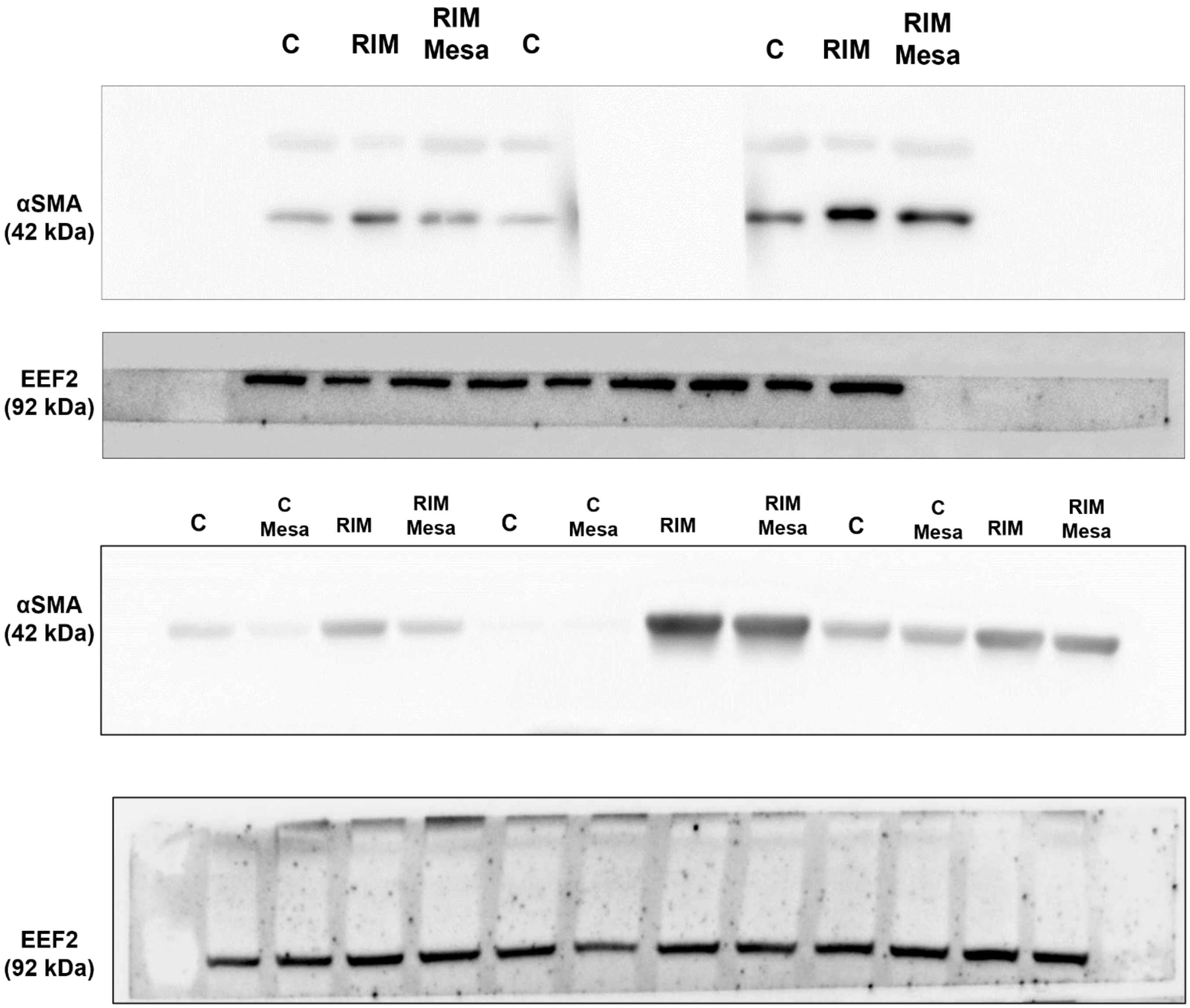

Original Western Blots for Figure 6 a

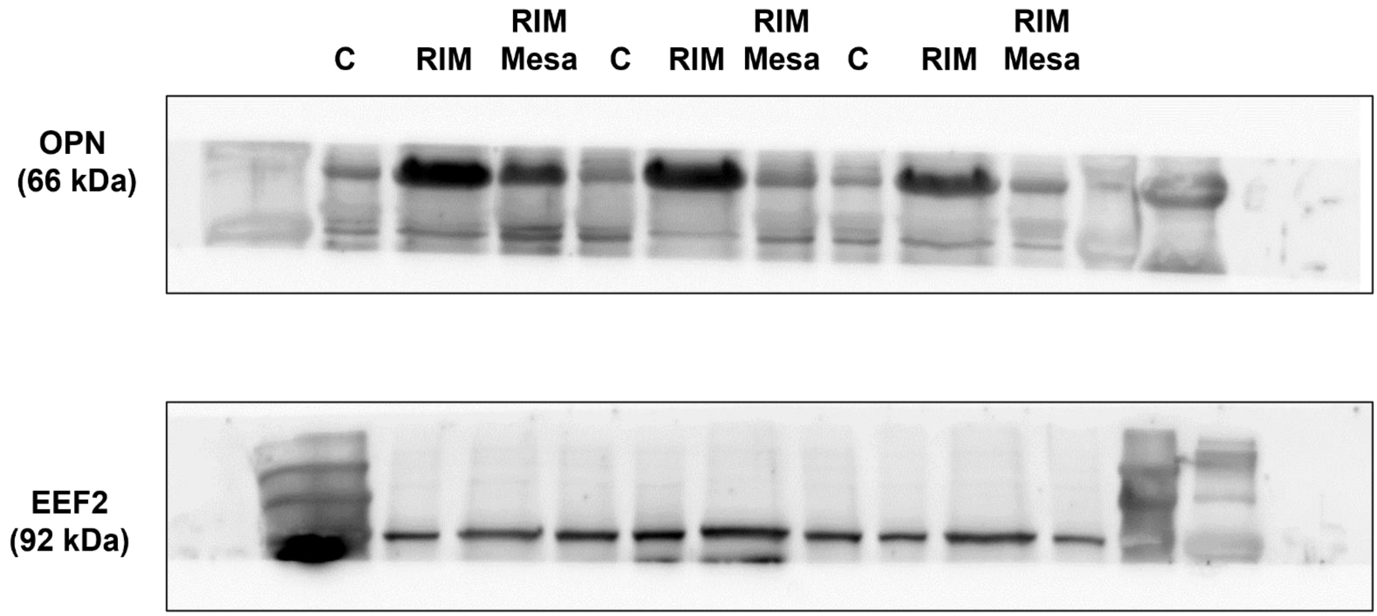

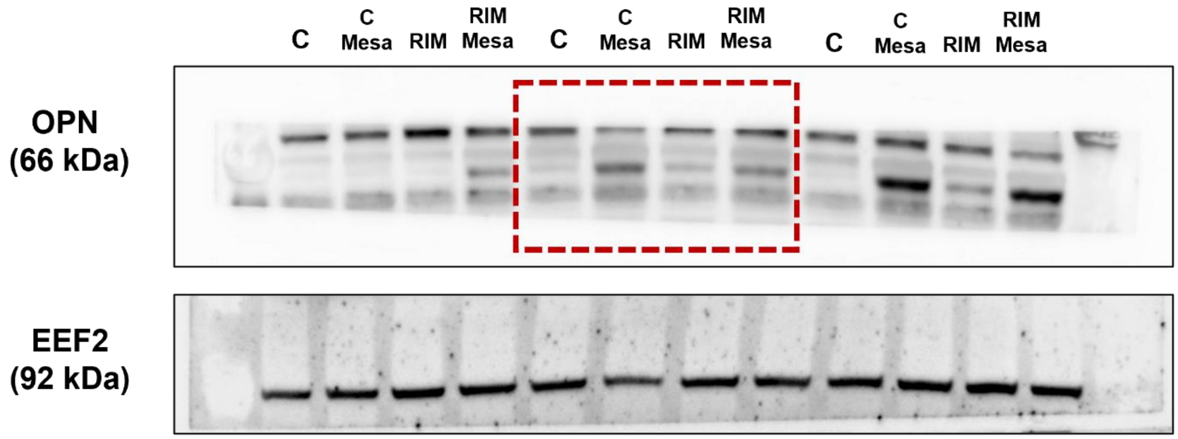

Information: The bands in the red marked area were excluded from analysis, because these bands were partially cut due to the slightly sloping running characteristics of the gel.

Original Western Blots for Figure 6 c

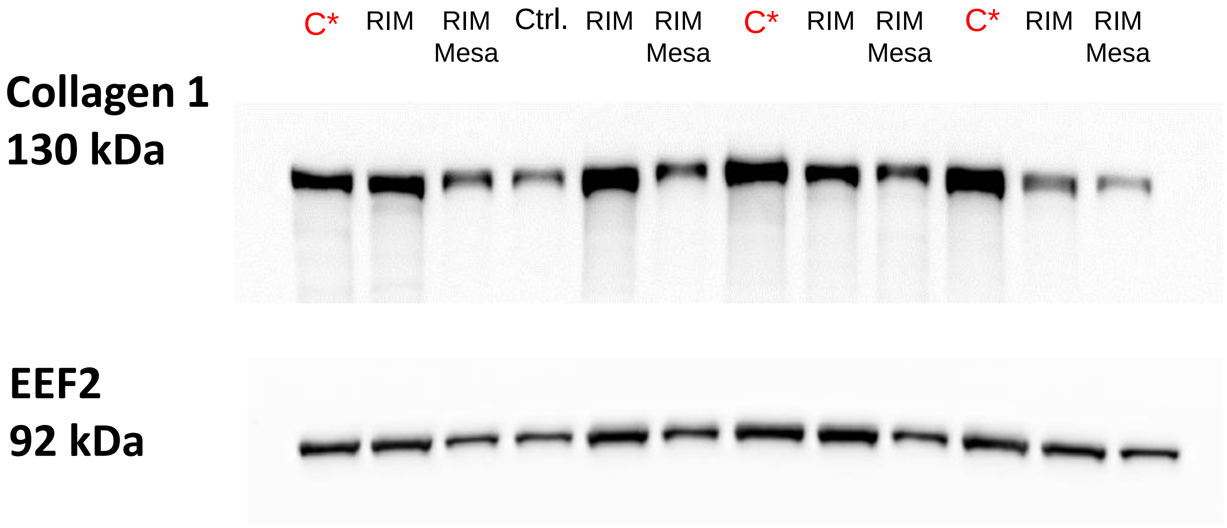

Information: This western blot was performed for the revision of the manuscript. Only 1 of the original controls used for the other experiments was still available (Ctrl.). Newly obtained control fibroblasts (C*) were isolated from male donors, which were not age-matched to the RIM samples. Therefore, Non-RIM fibroblasts were not included in the formal analysis of this experiment.
